# Allelic variation in rice *Fertilization Independent Endosperm 1* contributes to grain width under high night temperature stress

**DOI:** 10.1111/nph.16897

**Published:** 2020-09-23

**Authors:** Balpreet K. Dhatt, Puneet Paul, Jaspreet Sandhu, Waseem Hussain, Larissa Irvin, Feiyu Zhu, Maria Arlene Adviento‐Borbe, Argelia Lorence, Paul Staswick, Hongfeng Yu, Gota Morota, Harkamal Walia

**Affiliations:** ^1^ Department of Agronomy and Horticulture University of Nebraska‐Lincoln Lincoln NE 68583 USA; ^2^ Department of Computer Science and Engineering University of Nebraska‐Lincoln Lincoln NE 68588 USA; ^3^ Delta Water Management Research Unit USDA‐ARS Jonesboro AR 72401 USA; ^4^ Department of Chemistry and Physics Arkansas Biosciences Institute Arkansas State University Jonesboro AR 72467 USA; ^5^ Department of Animal and Poultry Sciences Virginia Polytechnic Institute and State University Blacksburg VA 24061 USA

**Keywords:** FIS‐PRC2, genome‐wide association analysis, grain development, grain quality, grain size, heat stress, rice, starch

## Abstract

- A higher minimum (night‐time) temperature is considered a greater limiting factor for reduced rice yield than a similar increase in maximum (daytime) temperature. While the physiological impact of high night temperature (HNT) has been studied, the genetic and molecular basis of HNT stress response remains unexplored.
- We examined the phenotypic variation for mature grain size (length and width) in a diverse set of rice accessions under HNT stress. Genome‐wide association analysis identified several HNT‐specific loci regulating grain size as well as loci that are common for optimal and HNT stress conditions.
- A novel locus contributing to grain width under HNT conditions colocalized with *Fie1*, a component of the FIS‐PRC2 complex. Our results suggest that the allelic difference controlling grain width under HNT is a result of differential transcript‐level response of *Fie1* in grains developing under HNT stress.
- We present evidence to support the role of *Fie1* in grain size regulation by testing overexpression (OE) and knockout mutants under heat stress. The OE mutants were either unaltered or had a positive impact on mature grain size under HNT, while the knockouts exhibited significant grain size reduction under these conditions.

## Introduction

Much of the extraordinary period of exponential crop productivity over the second half of the 20^th^ century is owed to the success of the Green Revolution (Wik *et al*., 2008; Pingali, 2012; Bailey‐Serres *et al*., 2019). Despite increasing land scarcity and rising population, the development of high‐yielding cultivars and improved agronomic practices have substantially decreased food deficits (Foley *et al*., 2011; Pingali, 2012). Although these improvements have reduced poverty and malnourishment, sustaining these gains will require even greater innovations to address the present‐day challenges in agriculture. In this context, climate change, especially the rising temperatures, threatens crop productivity (Porter & Gawith, 1999; Zhao *et al*., 2017). The global mean surface air temperature has increased by  0.5°C over the last century, which in part is a result of a faster increase in daily minimum temperatures (*T*_min_) compared with daily maximum temperatures (*T*_max_) (Karl *et al*., 1993; Easterling *et al*., 1997; Vose *et al*., 2005; Lobell, 2007; Thorne *et al*., 2016; Sun *et al*., 2019). Rice, which is a major source of calories and household income in many developing countries (Khush, 2005; Khush & Jena, 2009; Muthayya *et al*., 2014; Jagadish *et al*., 2015), is highly sensitive to increments in average minimum temperatures. Field‐level studies indicate a nearly 10% decline in grain yield for every 1°C increment in *T*_min_ (Peng *et al*., 2004; Lyman *et al*., 2013). An extensive regional (tropical/subtropical Asia) scale study suggested that rice productivity improved with a small increase in *T*_max_ but declined with higher *T*_min_, which is projected to have a net negative impact on the rice yield (Welch *et al*., 2010). Further, it has been shown that *T*_min_ has increased more sharply than *T*_max_ for some of the major rice‐growing regions of the world (Zhou *et al*., 2004; Padma Kumari *et al*., 2007).

Rice yield is a quantitative trait determined by the number of panicles, number of grains per panicle and grain weight (Sakamoto & Matsuoka, 2008; Xing & Zhang, 2010). Grain weight is predominantly determined by grain size – a function of grain length, width, and thickness as well as its degree of filling (Olsen, 2004; Hong *et al*., 2015). Genome‐wide association studies (GWAS) have identified major quantitative trait loci (QTLs) or genes regulating grain size under optimal conditions, for example *GRAIN SIZE* 2 (*GS2*), *GS3*, *GRAIN LENGTH AND WEIGHT 7* (*GLW7*), *GRAIN WEIGHT 6a* (*GW6a*), *GRAIN SHAPE 9* (*GS9*)*, GRAIN LENGTH 4* (*GL4*), *GRAIN SIZE ON CHROMOSOME 5* (*GSE5*), and *GRAIN WIDTH 8* (*GW8*) (Fan *et al*., 2006; Wang *et al*., 2012; Che *et al*., 2015; Duan *et al*., 2015, 2017; Hu *et al*., 2015; Song *et al*., 2015; Li *et al*., 2016; Si *et al*., 2016; Sun *et al*., 2016; Wu *et al*., 2017; Zhao *et al*., 2018). Several additional QTLs or genes involved in signaling (G‐proteins and mitogen‐activated protein kinases) and phytohormone homeostasis have also been reported to control grain size (Li *et al*., 2018, 2019 and references therein). Collectively, these studies have greatly advanced our understanding of rice grain size regulation. However, it is not clear if these genetic determinants will persist under higher temperatures predicted for many rice‐growing regions.

The effects of high day temperature (HDT) and high day‐night temperature (HDNT) on yield parameters are well studied. For instance, heat stress during reproductive development results in reduced seed set owing to decreased pollen viability (Prasad *et al*., 2006; Zinn *et al*., 2010; Bokszczanin & Fragkostefanakis, 2013; Hasanuzzaman *et al*., 2013; Jagadish *et al*., 2015; Fragkostefanakis *et al*., 2016; Röth *et al*., 2016; Arshad *et al*., 2017). Heat stress during early grain development alters the timing of endosperm cellularization (Folsom *et al*., 2014; Chen *et al*., 2016), while heat stress exposure during grain filling impacts rice grain size and quality (Lisle *et al*., 2000; Kadan *et al*., 2008; Fitzgerald *et al*., 2009; Sreenivasulu *et al*., 2015; Ali *et al*., 2019). Recently, it has been suggested that high night temperature (HNT) negatively impacts rice grain yield, primarily because of higher whole‐plant respiratory rates (Ziska & Manalo, 1996; Peng *et al*., 2004; Morita *et al*., 2005; Cheng *et al*., 2009; Ishimaru *et al*., 2009; Mohammed *et al*., 2013; Coast *et al*., 2015; Peraudeau *et al*., 2015; Bahuguna *et al*., 2017). Higher night temperatures can also alter the source‐to‐sink translocation of nitrogen and nonstructural carbohydrates, leading to reduced grain‐filling rates, thus influencing grain weight, width, and quality parameters (Shi *et al*., 2013). By contrast, our understanding of the genetic and molecular variation for HNT stress response in rice germplasm is largely unexplored. Therefore, we examined a diverse set of rice accessions from the rice diversity panel 1 (RDP1, Liakat Ali *et al*., 2011; Zhao *et al*., 2011; Eizenga *et al*., 2014) to identify loci controlling mature grain size under HNT by imposing a terminal HNT stress initiated just after fertilization. Here, we present results from two of the grain size determinants, length and width. GWAS revealed that *Fie1*, a component of the FIS‐PRC2 complex, regulates grain width under HNT but is not a significant source of variation under optimal night temperatures. We provide functional validation for the role of *Fie1* in grain size regulation using overexpression and knockout mutants under heat stress.

## Materials and Methods

### Plant material and growth conditions

We selected 273 accessions from the Rice Diversity Panel 1 (RDP1) corresponding to different subpopulations to screen for response to HNT stress (Table S1). Dehulled grains were sterilized with bleach (40% v/v) and water and germinated in dark on half‐strength Murashige & Skoog media. Six seedlings per accession were transplanted to pasteurized soil in 4 inch (101.6 mm) pots in a randomized complete block design. Plants were grown in a glasshouse under controlled conditions (16 h 30 ± 1°C : 8 h 23 ± 1°C, light : dark, relative humidity 55–60%). At 1 d after *c*. 50–70% of the primary panicle underwent flowering (Sandhu *et al*., 2019), three plants from each accession were transferred to a glasshouse under 16 h 30 ± 1°C : 8 h 28 ± 1°C, light : dark conditions for a terminal HNT treatment and the remaining three plants for each accession were maintained under the control glasshouse conditions (30 ± 1°C : 23 ± 1°C, light : dark). HNT stress and control conditions for this study represent the glasshouse air temperatures. All plants were harvested at physiological maturity. The primary panicles were tagged at flowering and harvested separately.

### Mature grain morphometric measurements and analysis

The harvested panicles were dried (30 ± 1°C) for 2 weeks. The dehulled mature grains from primary panicles were scanned using an Epson Expression 12000 XL (Epson America Inc., Los Alamitos, CA, USA) scanner at 600 dpi resolution (Dhatt *et al*., 2019). The scanned images were used to obtain morphometric measurements on mature grain size (length and width) using an inhouse developed matlab application (Zhu *et al*., 2020). The grain dimensions derived from the scanned grain images were checked for normality and outliers were removed. The mature grain size data were analyzed, and adjusted means for each accession across the replications were obtained using the following statistical model:

$$y_{\mathrm{ik}}=+g_{i}+r_{k}+\varepsilon_{\mathrm{ik}}$$

where $y_{\mathrm{ik}}$ refers to the performance of the *i*th accession in the *k*th replication, ***μ*** is the intercept, $g_{i}$ is the effect of the *i*th accession, $r_{k}$ is the effect of *k*th replication, and $\varepsilon_{\mathrm{ik}}$ is the residual error associated with the observation $y_{\mathrm{ik}}$. All analyses was performed in the R environment (R Core Team, 2019). Further, the adjusted means of each accession were used for GWAS.

### Genome‐wide association study (GWAS)

For GWAS analysis, a high‐density rice array (HDRA) of a 700k single nucleotide polymorphism (SNP) marker dataset was used (McCouch *et al*., 2016). After filtering for the missing data (< 20%) and minor allele frequency (< 5%), 411 066 SNPs were retained for GWAS. Before GWAS, principle component analysis (PCA) was performed (Zheng *et al*., 2012) to assess the population structure of the rice accessions (Fig. S1). Next, GWAS analysis was carried out in the R package rrblup (Endelman, 2011) using the following single marker linear mixed model:

y=1μ+Xβ+sα+Zg+ε

where, **y** is a vector of observations, μ is the overall mean, **X** is the design matrix for fixed effects, β is a vector of principle components accounting for population structure, s is a vector reflecting the number of alleles (0, 2) of each genotype at particular SNP locus, α is the effect of the SNP, Z is the design matrix for random effects, g is the vector of random effects accounting for relatedness and $g∼N\left(0,G\sigma_{g}^{2}\right)$; **G** is the genomic relationship matrix of the genotypes, $\sigma_{g}^{2}$ is the genetic variance, and ε is the vector of residuals. The outputs generated from GWAS analysis were used to plot the Q‐Q plots and Manhattan plots using the qqman package in r (Turner, 2014). The suggested threshold level of *P* < 3.3 × 10^−6^ or –log_10_(*P*) > 5.4 was used to declare the genome‐wide significance of SNP markers (Bai *et al*., 2016). Additionally, *R* ^2^‐values representing phenotypic variance contribution of each marker (or SNP) to the total variance were calculated using the bglr package (Pérez & De Los Campos, 2014). Narrow‐sense heritability (*h^2^*) of the lead SNP with or without accounting for linkage disequilibrium (LD) was estimated by jointly fitting the lead SNP along with all the other SNPs or fitting the lead SNP alone via a genomic restricted maximum likelihood method (Yang *et al*., 2017) using the R package sommer (Covarrubias‐Pazaran, 2016) as $h^{2}=\frac{\sigma_{g}^{2}}{\sigma_{g}^{2}+\sigma_{e}^{2}}$, where $\sigma_{g}^{2}$ is the genetic variance and $\sigma_{e}^{2}$ is the residual variance.

### Generation of transgenic plants and gene expression analysis

To generate CRISPR‐Cas9 mutants for *Fie1* (*Os08g04290*), single‐guide RNAs (sgRNAs) were designed using crispr‐P 1.0 (http://crispr.hzau.edu.cn/CRISPR/) (Lei *et al*., 2014). CRISPR‐Cas9 constructs were developed following the protocols described in Lowder *et al*. (2015). pYPQ141C vector was used to clone all sgRNAs, which were then transferred to a destination vector (pANIC6B) along with a vector containing Cas9 (pYPQ167) using LR clonase. The destination vector was transformed into *Agrobacterium tumifaciens*, which was further used to infect callii of Kitaake, a *temperate japonica* accession that carries a major allele for *Fie1* (Paul *et al*., 2020a). For *Fie1* knockout mutants, T1 segregates carrying mutations and lacking Cas9 (confirmed by β‐glucuronidase screening assay) were considered for downstream analysis. T0, T1, and T2 plants were screened for mutations by Sanger sequencing. T2 or later generations of mutants (*fie1^CR2^* and *fie1^CR3^*) were used in the study. Overexpression (OE) mutants (*Fie1^OE10^* and *Fie1^OE11^*) were considered from Folsom *et al*. (2014).

### Developing and mature grain analysis of *Fie1* mutants

To precisely assess the impact of high temperatures on *Fie1* mutants with respect to grains, florets marked at the time of fertilization (anthesis) were evaluated for downstream analysis: morphometric measurements of developing and mature grain and single‐grain weight at maturity. For developing grain analysis, florets were marked at the time of fertilization, and 1 d after fertilization (DAF), plants were subjected to HNT (30 ± 1°C : 28 ± 1°C, light : dark), HDNT (36 ± 1°C : 32 ± 1°C, light : dark) or constantly kept under control conditions (30 ± 1°C : 23 ± 1°C, light : dark). The marked florets from the respective temperature treatments were harvested at 4, 7, and 10 DAF. The images were processed using Imagej (Abramoff *et al*., 2004) to extract the developing grain length and width. For this, 15–20 marked florets from four plants per treatment per mutant line were evaluated. For analysis of mature grain traits, plants were subjected to HNT until maturity, HDNT from 1 to 10 DAF and moved back to control, or constantly kept under control conditions. The plants harvested at physiological maturity were used for downstream analysis. For this, 300–600 marked grains from 15–20 plants per treatment per mutant line were evaluated at maturity. For scanning electron microscopy (SEM), mature rice grains were processed as described in Dhatt *et al*. (2019).

### Genomic DNA and RNA extraction, RT‐qPCR, and DNA methylation assay

To screen for mutations in the knockouts, genomic DNA was isolated from leaves using the sucrose method (Berendzen *et al*., 2005). The region of interest was amplified using Kapa3G Plant PCR Kits (Kapa Biosystems, Wilmington, MA, USA) according to the manufacturer’s protocol. The amplicon was then sequenced for genotyping. RNA extraction and quantitative reverse transcription polymerase chain reaction (RT‐qPCR) were performed as described in Dhatt *et al*. (2019). Briefly, 1 µg of total RNA extracted from developing grains (4, 7 and 10 DAF) was used for cDNA synthesis. Gene‐specific primers were used for RT‐qPCR. Ubiquitin (*UBQ5*) gene was used as reference (Jain *et al*., 2006; Paul *et al*., 2020b). A minimum of two independent biological replicates and three technical replicates were used. The analysis was done using standard methods (Livak & Schmittgen, 2001) and plotted as log_2_(fold‐change) (Fragkostefanakis *et al*., 2015). DNA methylation assay was performed using the McrBC enzyme as described by Folsom *et al*. (2014). Primers used in the study are listed in Table S2.

## Results

### Phenotypic variation for grain size under HNT stress

To determine the extent of phenotypic variation in rice for grain size under heat stress, we imposed a terminal HNT treatment beginning 1 d after flowering and a corresponding control temperature treatment on 273 rice accessions from the rice diversity panel 1 (RDP1; Fig. S1; Table S1). The grains harvested at physiological maturity from the primary panicle were measured for length and width. Both these parameters were normally distributed and were hence directly accessible for downstream GWAS (Figs S2, S3). To gain an initial insight into the extent of phenotypic variation for HNT response in the diversity panel, we examined the accessions that exhibited high and low degrees of sensitivity to HNT stress by comparing the upper and lower tenth percentiles of accessions across grain length and width. For grain length, 13 and 19 accessions corresponded to upper and lower tenth percentiles, respectively, while 10 and 18 accessions corresponded to upper and lower tenth percentiles for grain width, respectively (Table S3). The sensitivity or tolerance was determined by the ratio of HNT and control for each of the parameters. We identified only three tolerant accessions that exhibited low sensitivity (range 1.10–1.17; HNT/control) for both mature grain length and width. Seven accessions had high sensitivity (range 0.85–0.94) for these two traits under HNT stress relative to control conditions (Table S3). This suggested that tolerance to HNT stress for grain length and width traits is probably determined independently in rice (Philipp *et al*., 2018) as there are very few accessions that can maintain both grain length and width under HNT stress conditions.

**Fig. 1:**
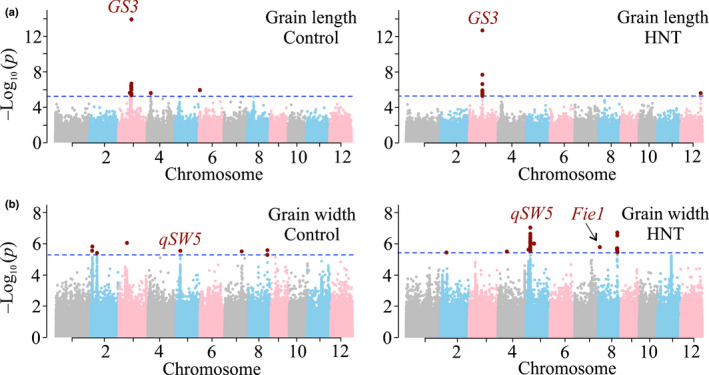
Manhattan plots of genome‐wide association results for rice mature grain length (a) and width (b) under control and high night temperature (HNT) stress conditions. The blue line indicates cutoff of significance threshold (*P* < 3.3 × 10^−6^ or −log_10_(*P*) > 5.4) level and significant single nucleotide polymorphism (SNPs) are highlighted with maroon dots. The previously known major grain size genes/quantitative trait loci (QTLs) (*GS3* and *qSW5*) under optimal (unstressed) conditions are labeled. *Fie1* (chr 8:2098482, SNP position) is a candidate gene for regulating phenotypic variation of grain width under HNT conditions in rice (indicated with an arrow).

Next, we aimed to elucidate the genetic basis of grain size variation under HNT stress and control conditions by performing independent genome‐wide association (GWA) analysis for mature grain length and width (Figs 1, S4). We identified a total of 63 significant SNPs associated with grain size under control (length = 13, width = 8) and HNT stress (length = 13, width = 29) conditions (Table S4). The most significant SNP for mature grain length was detected on chromosome (chr) 3 (chr3.16732086) under both control and HNT conditions (Fig. 1). The gene underlying this locus encodes *GS3*, a major regulator of grain size (Fan *et al*., 2006) and explained 13.24% and 12.71% of phenotypic variation under control and HNT conditions, respectively (Table S4). Further, heritability (*h* ^2^) for this SNP with and without correcting for linkage disequilibrium (LD) was 0.35 and 0.42 under control and 0.37 and 0.46 under HNT stress (Table S5). In addition, two significant SNPs (chr4.4655556 and chr6.1112028) for grain length were detected only under control conditions. The region spanning the SNP on chr 4 has previously been reported as *deformed interior floral organ 1*, which regulates rice reproductive development (Sun *et al*., 2017). The SNP on chr 6 is an expressed protein (*Os06g03030*). A single major locus on chr 12 (SNP: chr12.25310347) was only detected for mature grain length under HNT and explained 6.18% of phenotypic variation (Table S4). This SNP is in the intergenic region of two expressed proteins, *Os12g40930* and *Os12g40940*, which have low transcript abundance in developing seeds. This HNT‐specific locus is not known to be associated with rice grain length in other mapping studies under optimal temperatures.

**Fig. 2:**
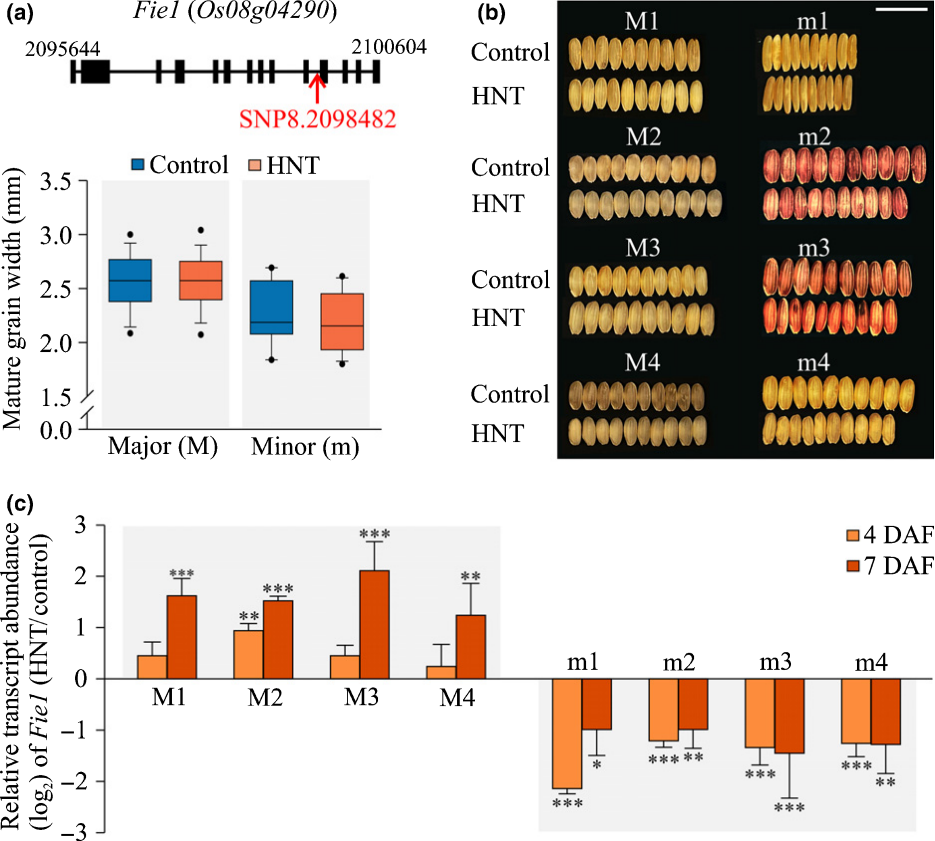
Characterization of *Fie1* locus as a determinant of grain width under high night temperature (HNT) stress in rice. (a) Upper panel: *Fie1* gene model with the significant single nucleotide polymorphism (SNP) position in the intron (exons, rectangle; intron, line). Lower panel: box plot showing the additive effect of *SNP8.2098482* under control and high night temperature (HNT) conditions; error bars represent ± SD. The SNP is significant (−log_10_(*P*) = 5.65) under HNT. The *x*‐axis shows the allelic groups, major (CC) and minor (GG), and the *y*‐axis shows grain width (mm). (b) Representative mature grain images of four major (denoted as ‘M’; M1–M4) and minor (denoted as ‘m’; m1–m4) allelic group accessions under control and HNT conditions (*n* = 10 grains). Images were digitally extracted and scaled for comparison (bar, 1 cm). (c) Relative transcript abundance of *Fie1* in the four major and minor allelic group accessions at 4 and 7 d after fertilization (DAF) under HNT conditions. Values were normalized against control for the respective time point. Error bars represent ± SD (*n* = 10–15 developing seeds per biological replicate; two biological and three technical replicates were used). Significant differences are depicted by asterisks (***, *P* < 0.001; **, *P* < 0.01; *, *P* < 0.05) based on a *t*‐test.

For grain width, we identified several SNPs that had a higher significance under HNT or were only detected under HNT conditions. For example, the peak on chr 5 had higher significance under the HNT condition (*P* < 6.87) compared with the control (*P* < 5.56). The lead SNP (chr5.5348012) underlying this peak explained 4.1% and 6.3% of phenotypic variation under control and HNT conditions, respectively (Table S4). The phenotypic variation explained by this lead SNP is similar to the phenotypic variation detected for the same SNP from previous studies (Huang *et al*., 2010; Zhao *et al*., 2011), and this SNP corresponds to *qSW5/GW5*, that is, a known regulator for grain width (Weng *et al*., 2008; Duan *et al*., 2017; Liu *et al*., 2017; Kumar *et al*., 2019). Another significant SNP for grain width that was stronger under HNT was detected on chr 8. The lead SNP (chr8.24070386) underlying this peak explained 5.7% of phenotypic variation and was in proximity with four genes (*Os08g37980*, *Os08g37990*, *Os08g38000* and *Os08g38010*) encoding three expressed/hypothetical genes and a retrotransposon (Tables S4, S6). A second significant SNP on chr 8 (SNP: chr8.2098482; *P* < 5.65) was associated with grain width under the HNT condition only (Fig. 1; Table S4). This SNP localizes to the intronic region of *Fertilization Independent Endosperm 1* (*Fie1*), which encodes for a protein component of the Polycomb Repressive Complex 2 (FIS‐PRC2; Figs 1, 2a). PRC2 is involved in endosperm development in plants and some of the processes regulated by PRC2 and *Fie1* are sensitive to heat stress (Folsom *et al*., 2014). Further, *h^2^* values for this SNP with and without correcting for LD were 0.19 and 0.31, respectively (Table S7). Accounting for LD removes confounding effects arising from the rest of genome‐wide markers and provides a more realistic contribution of *Fie1* SNP to the total genetic variation. Considering all these assessments, we reasoned that the *Fie1* locus is a promising candidate to examine further for genetic control of grain width under HNT stress.

### Allelic variation in *Fie1* may contribute to grain width under HNT stress

The two allelic groups at chr8.2098482 SNP located in *Fie1* differed in grain width under HNT stress and explained 4.88% of phenotypic variation (Fig. 2a; Tables S4, S7). The group of accessions (*n* = 190) exhibiting significantly higher mature grain width under HNT have the ‘CC’ allele, referred to as the major allele. The other group of accessions (*n* = 11) with lower mature grain width under HNT have ‘GG’ allele and is referred to as the minor allele (Fig. 2a,b). Although the mean value for grain width for the major allelic group was higher than that of the minor allelic group under control conditions, the difference was not statistically significant.

Rice *Fie1* is only expressed in developing endosperm from 4 to 10 DAF (Zhang *et al*., 2012) and its transcript abundance is altered in response to heat stress treatment (34°C : 29°C, light : dark; Folsom *et al*., 2014). Therefore, we investigated whether the *Fie1* transcript abundance is also sensitive to HNT stress alone, and if so, whether the allelic difference at the locus manifests through differential transcript abundance. For this, we randomly selected four accessions from each allelic group (Fig. 2b) and measured transcript abundances of *Fie1* under HNT stress conditions, as well as re‐evaluating mature grain size parameters in the respective accessions (Fig. 2b,c; Table S8). The transcript abundance of *Fie1* coincides with the early grain‐filling period in rice (Fig. S5). The major allelic group accessions (M1–M4) exhibited significantly higher transcript abundance of *Fie1* in developing grain at 7 DAF in response to HNT stress (Fig. 2c). By contrast, minor allelic group accessions (m1–m4) showed significantly lower *Fie1* transcript abundances at 4 and 7 DAF under HNT stress (Fig. 2c). This result supports our hypothesis that *Fie1* accounts for some of the genetic variation for grain width under HNT stress. As *Fie1* exhibits differential methylation status between leaves and developing seed tissue, we examined if the site of the lead SNP could be a casual SNP (CC vs GG allele) resulting in differential transcript abundance owing to presence of cytosine DNA methylation. We examined the methylation status of the region encompassing the *Fie1* SNP in the four major and minor allelic accessions and did not find evidence of DNA methylation at this SNP (Fig. S6). To assess whether variation in transcript abundance for nearby genes might be involved, we examined the expression level response of four additional genes in the two allelic groups under HNT (Fig. S7). We did not detect any variations in transcript abundance for three of the four neighboring genes (*Os08g04270*, *Os08g04280* and *Os08g04310*) in developing grains (4 and 7 DAF) of the two allelic groups under HNT stress (Fig. S7). One of the neighboring genes (*Os08g04300*) exhibited increased transcript abundance in developing grains (7 DAF) under HNT, but there was no difference in the response between the major and minor allelic accessions (Fig. S7). Overall, our results suggest that allelic difference in *Fie1* transcript abundance could be a determinant of grain width under the HNT condition.

### *Fie1* negatively regulates grain width under control conditions

**Fig. 3:**
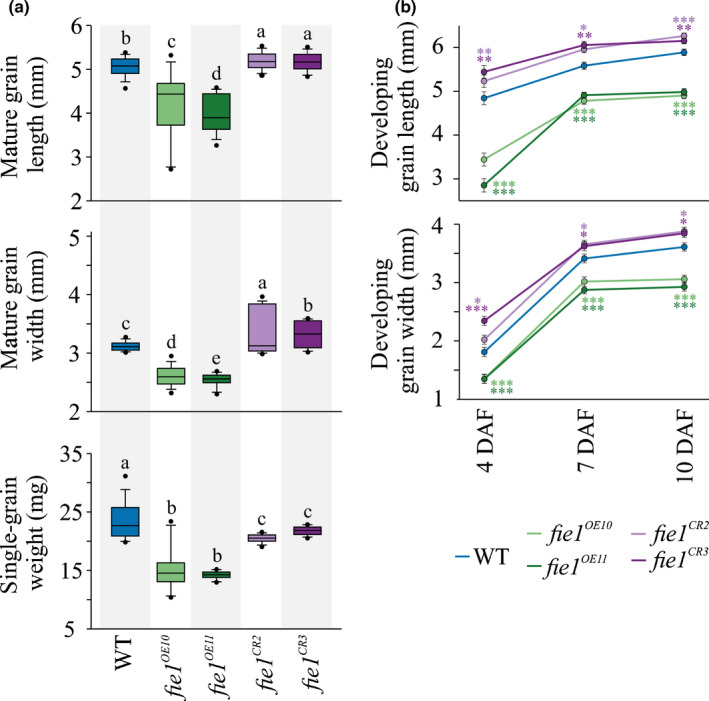
*Fie1* negatively regulates grain width under control conditions in rice. (a) Mature grain length, width, and single‐grain weight of wild‐type (WT), overexpression (*Fie1^OE10^* and *Fie1^OE11^*) and knockout mutants (*fie1^CR2^* and *fie1^CR3^*). Box plots show the median and the upper quartiles and black dots signify outliers. Significant differences (*P* < 0.05) are indicated by different letters based on a *t*‐test (*n* = 300–600 marked seeds from 15–20 plants). (b) Developing grain length and width of the mutants. Florets marked at the time of fertilization were collected at the respective developmental time points (4, 7 and 10 d after fertilization; DAF). Error bars represent ± SE. Significant differences are depicted by asterisks (***, *P* < 0.001; **, *P* < 0.01; *, *P* < 0.05) based on a *t*‐test (*n* = 15–20 marked developing grains from four plants per line).

To directly address whether *Fie1* abundance in developing grain regulates grain width under HNT stress, we generated OE lines and CRISPR‐Cas9 (CR)‐based knockout mutants in Kitaake, a *temperate japonica* cultivar, which naturally carries the major allele (CC) for SNP chr 8:2098482. Two homozygous knockout mutants (*fie1^CR2^* and *fie1^CR3^*) had 1 bp insertions in the targeted region and resulted in a premature stop codon, probably resulting in truncated proteins (Fig. S8). The OE (*fie1^OE10^* and *fie1^OE11^*) showed a markedly reduced plant height, while no vegetative stage phenotypic differences were observed for knockouts relative to the wild‐type (WT; Fig. S9). Under optimal growth conditions, the OE mutants exhibited a significant decrease in grain length and width compared with the WT, while knockouts showed a significant increase in these two parameters at maturity (Fig. 3a). These results are consistent with the observation of outer epidermal cells of mature grains via scanning electron microscopy (SEM), which show that the knockouts have increased cell width and length as compared with the WT under control conditions, while the OE mutants exhibited a decrease in the respective parameters (Table S9). Further, we observed a significant decline in single‐grain weight for both OE and knockout mutants at maturity; however, the reduction was more severe for OE mutants than for knockouts (Fig. 3a). As *Fie1* is preferentially expressed in developing grains (from 4 to 10 DAF; Fig. S5), we asked whether differences in grain length and width observed in mutants and OE lines at maturity are discernible in the developmental window that coincides with *Fie1* activity. For this, we recorded the growth dynamics of developing grains (4, 7 and 10 DAF) from the mutants under control conditions (Fig. 3b). We detected a significant decrease in developing grain length and width for OE mutants relative to the WT (Fig. 3b). By contrast, knockouts exhibited an increase in developing grain length and width relative to WT grains (Fig. 3b).

### Grain width in *Fie1* knockouts is sensitive to HNT

**Fig. 4:**
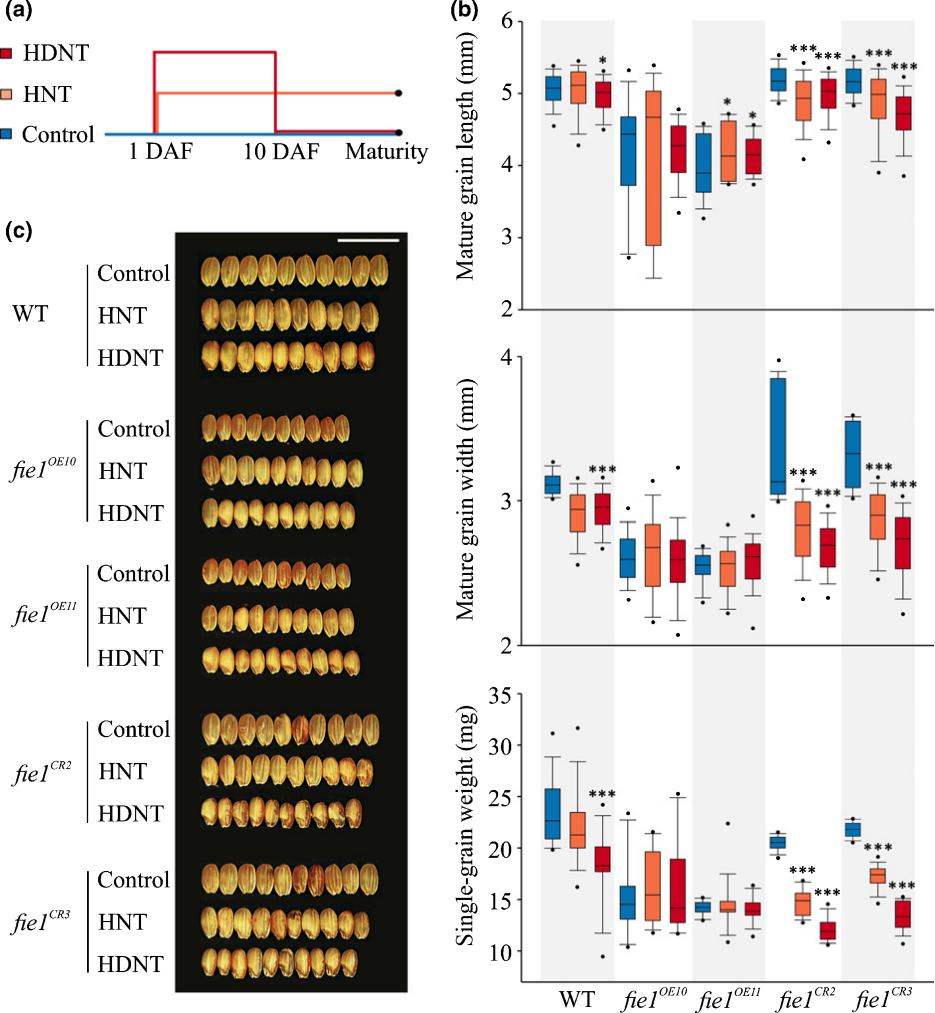
Grain width in *Fie1* knockouts in rice is sensitive to high night temperature (HNT). (a) Upper panel: pictogram illustrating heat stress regime. Florets were marked at the time of fertilization, and 1 d after fertilization (DAF) plants were subjected to either HNT (30 ± 1°C : 28 ± 1°C, , light : dark) until maturity, high day‐night temperature (HDNT; 36 ± 1°C : 32 ± 1°C, , light : dark) until 10 DAF and moved back to control (30 ± 1°C : 23 ± 1°C, light : dark) or constantly kept under control conditions. Plants were harvested at physiological maturity and the florets marked at the time of fertilization were considered for downstream analysis. (b) Mature grain length (top panel), width (middle panel), and single‐grain weight (bottom panel) of wild‐type (WT), overexpression (*fie1^OE10^* and *fie1^OE11^*), and knockout mutants (*fie1^CR2^* and *fie1^CR3^*) under control (blue colored), HNT (orange), and HDNT (red) conditions. Box plots show the median and the upper quartiles and black dots signify outliers (5^th^/95^th^ percentile). For statistics, a *t*‐test was used to compare HNT and HDNT with control (*n* = 300–600 marked grains from 15–20 plants per plant line per treatment). ***, *P* < 0.001; **, *P* < 0.01; * *P* < 0.05. (c) Representative mature seed images of WT and mutants under control, HNT and HDNT conditions. Images were digitally extracted and scaled for comparison (scale: 1 cm).

Next, we examined the phenotypic response of grains that are overabundant or deficient in *FIE1* under HNT stress conditions (Fig. 4). As average *T*_min_ is moderately and positively correlated with *T*_max_ for some of the major rice‐growing regions in Asia (Welch *et al*., 2010), we also included HDNT (36°C : 32°C, light : dark) stress treatment to determine the impact of *Fie1* misregulation on grain size under higher temperatures (Fig. 4). Unlike the terminal HNT stress treatment, HDNT stress is more severe, and hence it was only imposed from 1 to 10 DAF (Fig. 4a), which overlaps with *Fie1* transcriptional activity (Fig. S5). It is pertinent to point out that given the difference in intensity and duration of these two stress treatments, they are not directly comparable for discerning the impact of daytime higher temperature. The variation in means for WT mature grain length, width and single‐grain weight was not significant between the control and HNT (Fig. 4b). However, grain size, length and weight were significantly reduced when the WT plants were subjected to the HDNT stress treatment (Fig. 4b). We detected significant (*P* < 0.001) reduction in mature grain length, width and single‐grain weight for *fie1^CR2^* and *fie1^CR3^* subjected to HNT (until physiological maturity) and HDNT (from 1–10 DAF) compared with the control (Fig. 4b). This is consistent with our earlier finding that minor allele accessions with reduced *Fie1* transcript abundance had greater sensitivity to HNT stress. Collectively, our results suggest that grains that are deficient in *Fie1* are larger (although they weigh less) than WT grains under control conditions but are more sensitive to HNT stress with regard to grain size.

**Fig. 5:**
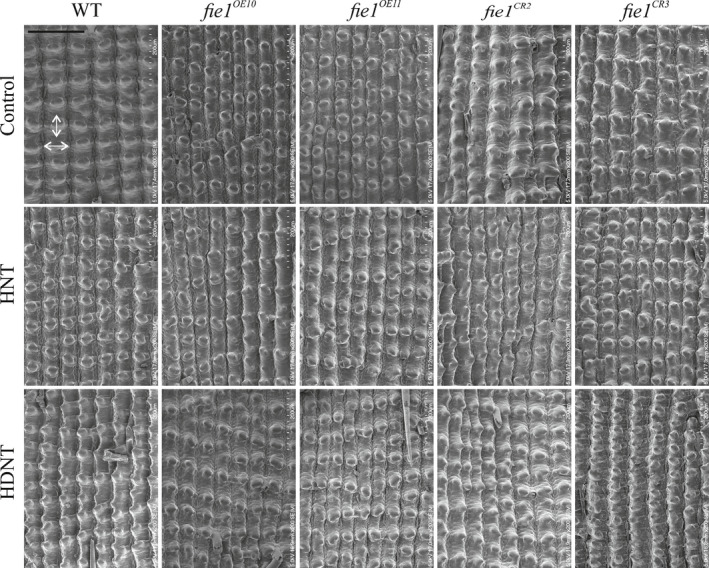
The outer epidermal surface of rice mature seeds from wild‐type (WT), overexpression (*fie1^OE10^* and *fie1^OE11^*) and knockout mutants (*fie1^CR2^* and *fie1^CR3^*) subjected to control, high night temperature (HNT), and high day‐night temperature (HDNT) stress (bar, 200 µm). Double‐headed white arrows indicate length and width of a representative single‐cell in a scanning electron microscopy image.

Only one of the two OE mutant line (*fie1^OE11^*) exhibited a significant (*P* < 0.05) increase in mature grain length subjected to higher temperatures (Fig. 5b). However, it should be noted that the control level for all these parameters in the OE lines was well below that of WT, possibly as a result of reduced plant stature in these lines. The OE mutant lines did not show any alteration with respect to mature grain width and single‐grain weight upon exposure to both heat treatments (Fig. 5b). Similarly, OE mutants were insensitive to heat treatments with respect to spikelet number and filled spikelet per panicle determined at the whole‐plant level (Table S10). We detected a similar outcome from SEM observations of the outer epidermal cells of the mature grain. While the knockouts and WT showed significant reduction in cell length and cell width of the outer epidermal cell on exposure to higher temperatures, no alterations were observed for OE mutants (Fig. 5; Table 1). Given the difference in seed size, we examined the transcript abundance of several known rice genes that positively (*BIG GRAIN 1*, *BG1*) or negatively (*GRAIN SIZE ON CHROMOSOME 5*, *GSE5*; *GRAIN LENGTH 7*, *GL7*; *SLENDER GRAIN*, *SLG*; *WIDE THICK GRAIN 1, WTG1*) regulate grain size. We observed that three out of the four negative regulators show higher transcript abundance in knockout mutants compared with the WT and OE mutants at 4, 7 and 10 DAF under higher temperatures (HNT and HDNT; Fig. S10). By contrast, *BG1* showed lower transcript abundance in the knockouts compared with the WT and OE mutants at 7 and 10 DAF under higher temperatures (Fig. S10). These results suggest that lower abundance of *Fie1* in developing grains under heat stress alters expression of several key rice grain size genes.

**Table 1:** Morphometric analysis of outer epidermal surface of rice mature seeds from wild‐type (WT), overexpression (*fie1^OE10^* and *fie1^OE11^*) and knockout mutants (*fie1^CR2^* and *fie1^CR3^*) subjected to control, high night temperature (HNT) and high day‐night temperature (HDNT) stress.

Line	Treatment	Total number of columns	Cells per column	Number of cells per unit area	Length (µm)	Width (µm)
WT	Control	6.33 ± 0.57	8.66 ± 0.51	54.83 ± 4.31	95.88 ± 11.06	74.26 ± 4.86
	HNT	7.41 ± 0.52	8.33 ± 0.51	61.75 ± 5.01	81.09 ± 8.65***	59.16 ± 5.04***
	HDNT	8.25 ± 0.95***	9.80 ± 1.12*	81.75 ± 15.96***	66.73 ± 8.58***	53.14 ± 5.71***
*fie1^OE10^*	Control	8.83 ± 0.28	9.33 ± 0.51	82.5 ± 5.19	69.38 ± 8.62	46.53 ± 5.28
	HNT	8.62 ± 0.47	8.87 ± 0.83	76.56 ± 7.99	78.14 ± 16.11**	46.85 ± 4.48
	HDNT	8.50 ± 0.57	8.62 ± 0.51	73.25 ± 5.50	69.79 ± 7.97	48.03 ± 4.45
*fie1^OE11^*	Control	8.00 ± 0.00	8.00 ± 0.00	64.00 ± 0.00	75.78 ± 9.26	51.61 ± 5.29
	HNT	8.50 ± 0.57	8.12 ± 0.35	69.12 ± 6.19	76.86 ± 17.86	51.94 ± 10.81
	HDNT	8.25 ± 0.95	8.50 ± 0.75	69.75 ± 5.60	77.42 ± 13.63	50.30 ± 11.12
*fie1^CR2^*	Control	5.87 ± 0.25	8.00 ± 0.00	47.00 ± 2.00	103.67 ± 6.54	80.09 ± 5.28
	HNT	8.06 ± 0.12***	8.50 ± 0.53	68.53 ± 3.43**	76.55 ± 7.91***	51.27 ± 4.50***
	HDNT	7.75 ± 0.5***	10.50 ± 0.75***	81.25 ± 5.73***	74.05 ± 7.81***	56.46 ± 4.20***
*fie1^CR3^*	Control	6.41 ± 0.52	7.83 ± 0.40	50.16 ± 2.25	103.61 ± 8.01	80.09 ± 6.16
	HNT	7.50 ± 0.43*	10.33 ± 1.03**	77.66 ± 9.65***	73.34 ± 10.31***	52.74 ± 5.39***
	HDNT	6.12 ± 0.25	11.25 ± 1.90***	69.25 ± 15.37**	76.74 ± 13.87***	62.96 ± 4.92***

Total number of columns, cells per column and number of cells per unit area were quantified from three biological replicates. The cell length and width of outer epidermal cells were quantified from 10 random cells per biological replicate. For statistics, Student’s *t*‐test was used to compare control vs HNT and control vs HDNT conditions for the respective plant line (± SD; *, *P* < 0.05; **, *P* < 0.01; ***, *P* < 0.001).

**Fig. 6:**
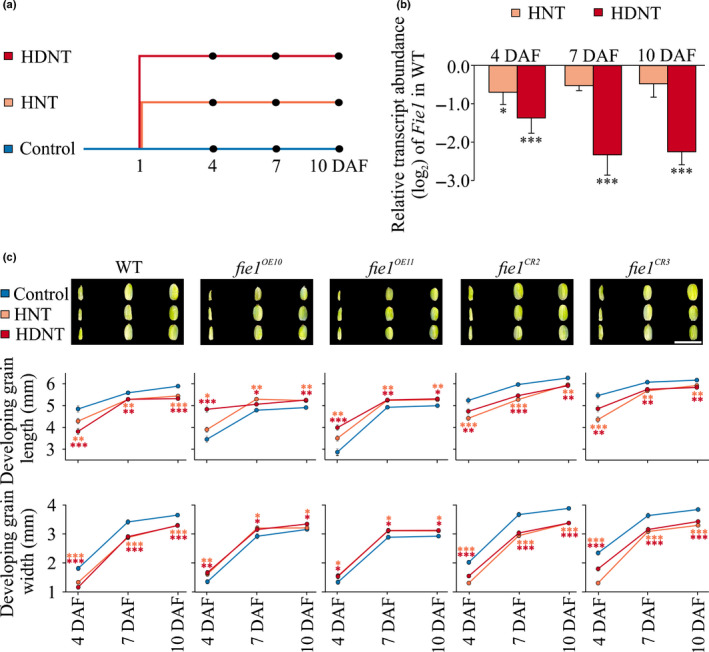
Developing grains of *Fie1* knockouts are sensitive to high night temperature (HNT) in rice. (a) Pictogram illustrating heat stress regime. Florets were marked at the time of fertilization, and at 1 d after fertilization (DAF) plants were subjected to either HNT (30 ± 1°C : 28 ± 1°C, light : dark), high day‐night temperature (HDNT; 36 ± 1°C : 32 ± 1°C, light : dark) or constantly kept under control conditions (30 ± 1°C : 23 ± 1°C, light : dark). The marked florets were harvested at 4, 7 and 10 DAF (marked with black dots) for downstream analysis. (b) Relative transcript abundance of *Fie1* in the wild‐type (WT) at 4, 7 and 10 DAF under HNT and HDNT conditions. Values were normalized against control for the respective time point. Error bars represent ± SD. For statistics, a *t*‐test was used: ***, *P* < 0.001; *, *P* < 0.05. (c) Top panel: representative images of developing grain (4, 7 and 10 DAF) of WT, overexpression (*fie1^OE10^* and *fie1^OE11^*) and knockout mutants (*fie1^CR2^* and *fie1^CR3^*) under control, HNT, and HDNT conditions. Images were digitally extracted and scaled for comparison (bar, 1 cm). Developing grain length (middle panel) and width (bottom panel) of the mutants. Error bars represent ± SE. For statistics, a *t*‐test was used to compare HNT and HDNT to the control (*n* = 15–20 marked developing seeds from four plants per treatment per line). ***, *P* < 0.001; **, *P* < 0.01; *, *P* < 0.05.

The contrasting response of OE and knockout *Fie1* mutants on mature grain phenotypes in response to heat stress led us to investigate its effect on earlier stages of grain development (Fig. 6). For this, we examined the *Fie1* transcript abundances at 4, 7, and 10 DAF under HNT and HDNT in WT grains (Fig. 6a,b). The *Fie1* transcript abundance at 4 DAF declines significantly under HNT as well as HDNT conditions relative to control (Fig. 6b). At 7 and 10 DAF, the *Fie1* transcript abundance was significantly lower under HDNT than under control conditions, whereas it did not show any alteration under HNT at the 7 and 10 DAF time points. This suggested that the molecular response of *Fie1* to HNT and HDNT stress may be distinct over a temporal scale. We next measured the length and width of developing grains corresponding to 4, 7 and 10 DAF, a developmental window that coincides with *Fie1* transcript abundance (Fig. S5). Consistent with the mature grain phenotype, WT and knockouts exhibited thermal sensitivity as evident from the reduced developing grain length and width under HNT and HDNT relative to control (Fig. 6c). On the other hand, *fie1^OE10^* and *fie1^OE11^* mutants showed a significant increase in developing grain length and width under higher temperatures compared with the control. Collectively, these results suggest that the grains deficient in *Fie1* exhibit increased sensitivity for decrease in grain size and weight under high temperatures, thus further supporting our hypothesis that higher susceptibility of minor allelic group to HNT stress could possibly be a result of lower transcript abundance of *Fie1* under HNT compared with the major allelic group.

### High night temperatures deteriorate grain quality in grains deficient in *Fie1*

**Fig. 7:**
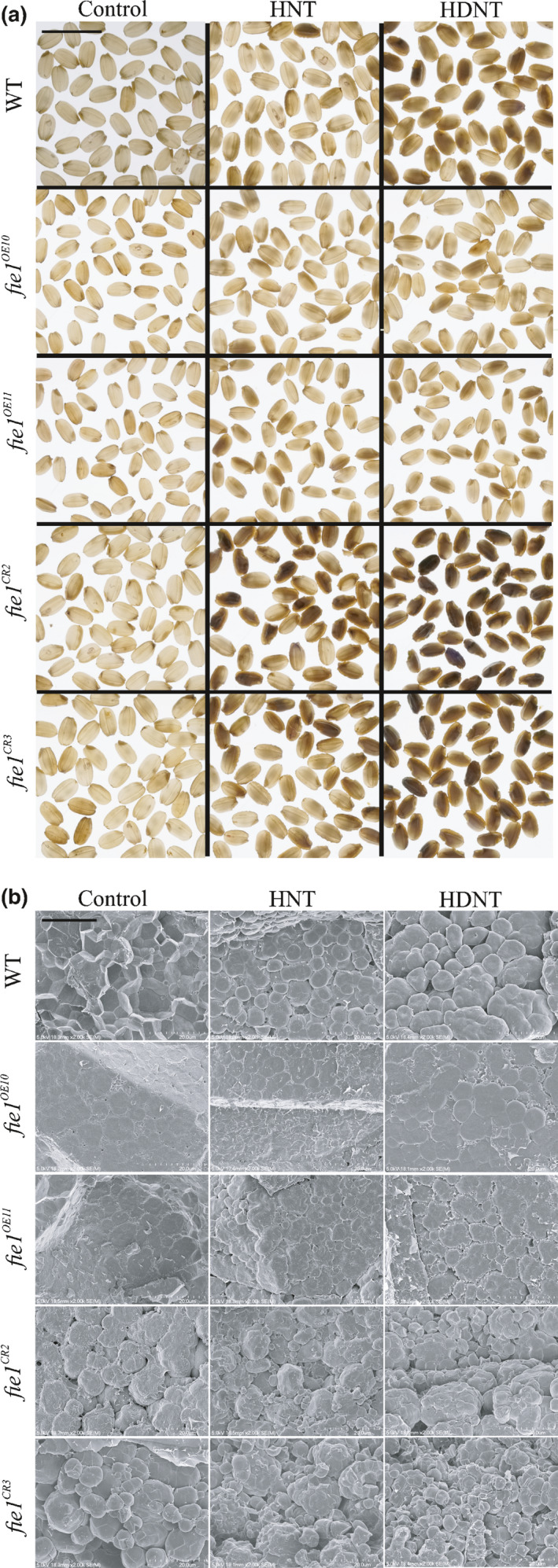
Misregulation of *Fie1* alters starch quality under heat stress in rice. (a) Representative light‐box images of 50 mature grains from wild‐type (WT), overexpression (*fie1^OE10^* and *fie1^OE11^*) and knockout mutants (*fie1^CR2^* and *fie1^CR3^*) subjected to control, high night temperature (HNT) and high day‐night temperature (HDNT) during grain development (bar, 1 cm). (b) Cross‐sections of mature grain of WT and mutants subjected to control, HNT and HDNT conditions observed via scanning electron microscopy (bar, 20 µm).

Exposure to high temperatures during grain development greatly increases chalkiness in rice (Tashiro & Wardlaw, 1991; Lisle *et al*., 2000; Tsutsui *et al*., 2013). Chalkiness is often a result of loosely packed starch granules and abnormal protein bodies (Wang *et al*., 2010) in the endosperm. Loose packing creates more air space, resulting in decreased transmittance of light. We observed increased chalkiness in *Fie1* knockouts when subjected to either HNT or HDNT (Fig. 7a). Further inspection of mature rice grains of the *Fie1* mutants via SEM revealed structural abnormalities in starch granules. Less tightly packed starch grains with a more rounded appearance were seen in WT following HNT and HDNT treatment (Fig. 7b). Mutant grains exhibited similar aberrations in starch structure even under control treatment. These observations prompted us to investigate transcript abundance of a selected set of genes known to be involved in the starch biosynthesis pathway in the developing grains (4, 7 and 10 DAF) of mutants subjected to higher temperature treatments (HNT and HDNT; Fig. S11). We analyzed six genes including the two subunits of ADP glucose pyrophosphorylase (AGPase; *AGPS2b* and *AGPL2a*), granule bound starch synthase (*GBSSI*) that regulates amylose biosynthesis (*Wx*), two starch synthase genes (*SSIIa* and *SSIVb*) involved in amylopectin biosynthesis, and rice basic leucine zipper (*RISBZ1/ bZIP58*) which regulates starch synthesis genes. We detected higher transcript abundance for all the tested genes in developing grains of mutants (both OE and knockouts) under control conditions (Fig. S11a). Developing grains of knockouts exhibited a general trend of reduced transcript abundances for most of the tested genes at all three developmental time points under heat stress (HNT and HDNT); however, the reduction was much more severe under HDNT conditions (Fig. S11b). Interestingly, grains from the OE mutants developing under HNT stress showed lower transcript abundances at 4 and 7 DAF, while their transcript repression was relieved at 10 DAF for four of the six tested genes (Fig. S11b).

We next examined whether the loose starch packaging associated with *Fie1* knockouts is also observed in the minor allele accessions under stress, given that minor allele accessions have lower *Fie1* transcript abundance relative to major allele accessions under HNT conditions (Fig. 2). Our SEM scans indicate that three of the four minor allele accessions have abnormal starch packing when exposed to a terminal HNT stress (Fig. S12). The four major and one minor allele accessions did not have an obvious starch packaging defect (Fig. S12). These results suggest that the allelic variation associated with *Fie1*, which results in differential transcript abundance, could be a contributing factor in determining chalkiness under HNT stress conditions.

## Discussion

We screened a large set of rice accessions to elucidate the extent of natural variation for impact of HNT stress on grain size (length and width). Our analysis suggests that the genetic basis of HNT sensitivity of grain length and width may not be determined by common regulators, as there are very few accessions where both grain length and width are highly sensitive or tolerant to HNT stress. Rice grain length is primarily determined during early grain development and probably earlier than grain width (Lizana *et al*., 2010; Pielot *et al*., 2015). It is therefore expected that the genetic determinants of these two traits have distinct temperature sensitivity across a diverse population. Our GWA analysis identified several novel loci regulating mature grain length and width under control and HNT conditions (Fig. 1; Table S4). We also identified several previously determined loci (*GS3* and *qSW5*) regulating these traits (Fan *et al*., 2006; Shomura *et al*., 2008; Weng *et al*., 2008; Huang *et al*., 2010; Mao *et al*., 2010; Zhao *et al*., 2011; Duan *et al*., 2017; Liu *et al*., 2017), thus validating our experimental setup and analysis. About 10 loci (*GS3*, *qSW5*, and others) were identified as significant contributors for grain size traits under both temperature regimes in our analysis. Some loci, such as ch12:25310347 for grain length, are unique to HNT stress conditions (Fig. 1). Another notable SNP (chr8:2098482) colocalized with the *Fie1*, which is identified as a significant contributor to grain width under HNT stress only (Fig. 1). The major allele for the SNP colocalizing with *Fie1* is highly prevalent in the diversity panel assayed in this study, indicating that the favorable allele is predominant in rice germplasm. This is not surprising, given the undesirable starch structure observed in some of the minor allele accessions under higher temperatures (Fig. S12). This inferior grain trait has probably undergone negative selection in the rice germplasm, resulting in selection against the minor allele.

*Fie1* encodes a component of the FIS‐PRC2 complex in rice and is related to the duplicated gene *Fie2*, which is a core component of the FIS‐PRC2 complex. Unlike *Fie1*, *Fie2* is expressed in most plant developmental stages, including grain development (Luo *et al*., 2009; Zhang *et al*., 2012; Huang *et al*., 2016). Although, there are several instances of allelic variation in epigenetic modifications (e.g. differential methylation) underlying variation for agronomic traits (Shindo *et al*., 2006; Shen *et al*., 2014; Fang *et al*., 2016), we present evidence of allelic variation associated with the *Fie1* as a contributing factor in grain size and quality determination in rice under high temperatures. *Fie1* is preferentially expressed in endosperm (between 4 and 10 DAF; Fig. S5) and its transcript abundance in developing grains is highly sensitive to incremental changes in temperature (Folsom *et al*., 2014). The phenotypic variation observed for mature grain width in response to HNT is associated with a polymorphism detected in the *Fie1* intron, although we have no evidence that this is a causal SNP (Fig. 2a,b). The major accessions with the ‘CC’ allele (chr 8, location 2099480) tend to have higher grain width under HNT, while minor allele accessions with the ‘GG’ allele are generally associated with narrower grains under HNT (Fig. 2a). Interestingly, two of the four tested minor allele accessions (m2, m3) have red pericarp. Red pericarp in rice is linked to a 14 bp mutation in the *Rc* gene (Sweeney *et al*., 2006). Hence, we wondered if the *Fie1* allelic variation could be linked to the red pericarp. A direct link between the minor allele for *Fie1* and red pericarp is unlikely as *Fie1* mutants do not have altered seed color. Further, a recent work where the *Rc* gene is functionally restored reported that grain size is not altered (Zhu *et al*., 2019).

The allelic difference in the grain width appears to be a result of contrasting transcript abundance response of *Fie1* in developing grains under HNT at 7 DAF (Fig. 2c). In this context, the four accessions tested with higher (major allelic accessions) and lower (minor allelic accessions) transcript abundance of *Fie1* in developing grains under HNT could be contributing to wider and narrower mature grains, respectively (Fig. 2). It is noteworthy that at 4 DAF, the expression level response of *Fie1* to HNT stress was highest for the major accession M2, which exhibited the greatest grain width under HNT, and was lowest for m1, which had the most slender grains among the examined accessions (Fig. 2c; Table S8). Overall, our expression analysis indicated that transcript abundance of *Fie1* in developing grains is likely to be a contributing factor in determining grain width under HNT stress.

We directly tested this hypothesis with mutants (OE, *fie1^OE10^* and *fie1^OE11^*; knockouts, *fie1^CR2^* and *fie1^CR3^*) misregulating *Fie1* abundance (Fig. S8). Knockouts should be specifically affected in grain development, as *Fie1* expression is narrowly limited to this developmental stage. However, OE lines were generated with the ubiquitin promoter, resulting in expression throughout the plant. This was evident from the markedly decreased stature of OE plants in the absence of any heat treatment. Under control conditions, the mutants possessing overabundance of *Fie1* transcripts showed a significant decrease in mature and developing grain length and width (Fig. 3). These results are in accordance with the previous findings that have found the smaller grain size in OE mutants to be partly a result of precocious endosperm cellularization (Folsom *et al*., 2014). Furthermore, the mutants deficient in *Fie1* exhibited a small but significant increase in mature and developing grain length and width under control conditions (Fig. 3) suggesting that *Fie1* might act as a negative regulator of grain size under control conditions. As the GWAS revealed *Fie1* as a potential candidate regulating grain width under HNT, we investigated the impact of HNT as well as HDNT on *Fie1* mutants (Figs 4, 5, 6). The grains deficient in *Fie1* exhibited higher thermal sensitivity, as evidenced by significant reduction in mature grain length, width and single‐grain weight, as well as cell width of the outer epidermal cells (Figs 4, 5; Table 1). By contrast, the grains overexpressing *Fie1* were mostly unaltered, although one line had a small positive impact on mature grain length under HNT and HDNT (Fig. 4b). Likewise, the developing grains from the *Fie1*‐deficient mutants showed reduced length and width at all three time points of early grain development (Fig. 6c). This indicates a critical role for *Fie1* acting early during grain development to maintain mature grain size under heat stress. Although overabundance of *Fie1* transcripts in the OE mutants exhibited a significant increase in developing grain length and width under HNT and HDNT conditions relative to the control, this did not carry through to the mature grains in most cases. This may be related to the fact that OE plants were smaller in stature and were possibly compromised in their ability to supply resources to grains throughout their development in comparison to control plants. Also, the WT Kitaake variety used for transformation already has the ‘CC’ major allele, which may have maximized the benefit from *Fie1* under these conditions. The increase in mature grain size and size of outer epidermal cells in the knockout mutants under optimal conditions (Fig. 3; Table S9) could be a consequence of a longer period of cellular expansion of seeds and nuclear proliferation, respectively, in seeds that are deficient in *Fie1*, a member of the PRC2 complex in rice. The PRC2 complex is required for timely initiation of endosperm cellularization, and *Fie1* transcript starts to accumulate just as the endosperm initiates cellularization (Gehring, 2013; Pires, 2014). Therefore, *Fie1* deficiency could delay this PRC2‐mediated endosperm transition. Delayed cellularization is also associated with increased auxin concentrations in Arabidopsis (Figueiredo *et al*., 2016; Batista *et al*., 2019). It is feasible that higher auxin concentrations in *Fie1*‐deficient, overproliferating coenocytic endosperm inhibit cell wall formation and increase auxin transport to seed coat tissue, resulting in cellular expansion and hence larger cell size (Batista *et al*., 2019; Paul *et al*., 2020a). The potential mechanistic link between *Fie1* and auxin accumulation and transport needs further examination. Similarly, the potential molecular association between reduced grain size of *Fie1* knockouts under heat stress and transcript abundance of grain size regulators needs to be explored in future studies.

It is noteworthy that even though *Fie1* knockouts have larger grains, their grain weight under control conditions is lower relative to the WT. We reasoned that it could be a result of less dense packaging of the starch in the knockout endosperm (Fig. 7). The striking difference in packaging density in the knockouts relative to the WT, and to a considerable degree for OE mutants (Fig. 7), is consistent with greater grain size and lower grain weight (Fig. 3). Further, gene expression analysis in the developing grains exposed to HNT and HDNT suggested that the observed chalky phenotype is associated with misregulation of starch biosynthesis genes (Fig. S11). Chalkiness in rice is also associated with nitrogen status of the seeds (Wada *et al*., 2019). It remains to be determined if some of the *Fie1* mutant phenotypes under heat stress could be ameliorated by supplemental nitrogen. Collectively, these data suggest that the early endosperm development events in response to heat stress likely have downstream impact on starch accumulation, packaging, and overall grain quality besides decreasing grain size.

### Conclusion

Several studies exploring natural variation in the regulation of important agronomic traits to improve rice grain yield and quality have been conducted. However, these studies have largely focused on testing the performance of the identified alleles under optimal conditions. The impact of these alleles under suboptimal temperatures has not been determined. Here, we show that for two grain size traits, some of the allelic variation is persistent across optimal and HNT stress conditions, while other novel alleles are specific for HNT conditions. We present evidence for one such variant within *Fie1*, which is probably associated with grain width under HNT stress. Although differential methylation at DNA and histone level has been reported to be the basis for variation of some agronomic traits, there are not many instances reported for allelic variation associated with an epigenetic regulator itself which results in phenotypic variation for agronomic traits. Our work suggests that *Fie1* is one such regulator. The mechanistic basis of differential transcript abundance of *Fie1* among the two allelic groups remains to be elucidated. Based on findings from our work and previous reports, it is possible that *Fie1* function diverged following duplication from *Fie2* to specifically enhance reproductive success in a range of environments with varying temperatures. As higher dosage of *Fie1* could potentially stabilize grain size and grain quality across a wider temperature range, it was probably selected by farmers and/or breeders across multiple environments, and hence the observed predominance of the favorable major allele in rice germplasm. Future work will also focus on deciphering the role of uncharacterized novel loci identified in this study for their role in the regulation of grain yield‐related parameters under heat stress.

## Author contributions

HW conceptualized and designed the project. PP led the study. PP, JS, BKD, LI performed the heat stress experiment on rice accessions. PP scanned mature grains for downstream analysis and FZ and HY analyzed these scanned images. WH and GM performed GWAS. HW and PP performed post‐GWAS analysis. BKD and JS generated *Fie1* mutants. BKD performed experiments on *Fie1* mutants. BKD and PP analyzed the results from the mutants. PP and HW wrote the manuscript. PS, AL and MAAB critically reviewed and analyzed the work. All authors read and approved the manuscript. BKD, PP, JS and WH contributed equally to this work.

## Supporting information


**Fig. S1** Principal component analysis (PCA) showing the population structure of the rice diversity association panel (RDP1) used in this study.
**Fig. S2** Phenotypic distribution of rice mature grain length and width under control and high night temperature (HNT) stress.
**Fig. S3** Rice subpopulation‐level phenotypic distribution of mature grain length and width under control and high night temperature (HNT) stress.
**Fig. S4** Q‐Q plots of –log_10_(*P*) values obtained from the linear mixed model for mature grain length and width under control and high night temperature (HNT) stress in rice.
**Fig. S5** Relative transcript abundance of *Fie1* in wild‐type (WT) developing seeds (4, 7, and 10 d after fertilization; DAF) under control conditions in rice.
**Fig. S6** DNA methylation analysis.
**Fig. S7** Relative transcript abundance of *Fie1* neighboring genes (two upstream and two downstream) in developing seeds in rice.
**Fig. S8** The mutants used in the study: *Fie1* overexpression (a; *fie1^OE10^* and *fie1^OE11^*) and knockouts (b; *fie1^CR2^* and *fie1^CR3^*) in rice.
**Fig. S9** Representative images of wild‐type (WT), knockout (*fie1^CR2^* and *fie1^CR3^*), and overexpression (*fie1^OE10^* and *fie1^OE11^*) mutants at day 65 in rice.
**Fig. S10** RT‐qPCR analysis of rice grain size‐related genes.
**Fig. S11** RT‐qPCR analysis for selected set of rice starch biosynthesis genes in the mutants.
**Fig. S12** Cross‐sections of mature grains from four major (M1–M4) and four minor (m1–m4) allelic accessions under HNT observed via scanning electron microscopy.

**Table S1** Rice accessions used in this study.
**Table S2** List of primers used in the study.
**Table S3** Upper and lower 10^th^ percentiles of accessions with respect to grain length and width based on ratio of HNT to control.
**Table S4** Significant SNPs associated with rice mature grain length and width under control and HNT.
**Table S5** Narrow‐sense heritability (*h* ^2^) of the top significant SNP with and without accounting for linkage disequilibrium for grain length and width under control and HNT conditions.
**Table S6** Gene expression values of the genes close to the other lead SNP on chromosome 8 (SNP8.24070386) based on different public datasets. Color shading is just to represent three different datasets used to evaluate the gene expression analysis.
**Table S7** Cumulative phenotypic variation of all the significant SNPs and the variation contribution of the top significant SNP associated with grain length and width in control and HNT conditions.
**Table S8** Four major (denoted by ‘M’) and minor (denoted by ‘m’) allele accessions corresponding to *Fie1* SNP (chr8.2098482) were re‐evaluated for mature grain length, width and single‐grain weight (SGW) under control and HNT conditions.
**Table S9** Morphometric analysis of outer epidermal surface of mature seeds from wild‐type (WT), overexpression (*fie1^OE10^* and *fie1^OE11^*) and knockout mutants (*fie1^CR2^* and *fie1^CR3^*) under control conditions.
**Table S10** Yield‐related parameters in WT and *Fie1* overexpression (OE) and knockout (CR) mutants under control, HNT, and high day‐night temperature (HDNT) treatments at the whole‐plant level.
Please note: Wiley Blackwell are not responsible for the content or functionality of any Supporting Information supplied by the authors. Any queries (other than missing material) should be directed to the *New Phytologist* Central Office.

## References

[nph16897-bib-0001] Abramoff M , Magalhães P , Sunanda RJ . 2004 Image processing with ImageJ. Biophotonics International 11: 36–42.

[nph16897-bib-0002] Ali F , Waters DLE , Ovenden B , Bundock P , Raymond CA , Rose TJ . 2019 Australian rice varieties vary in grain yield response to heat stress during reproductive and grain filling stages. Journal of Agronomy and Crop Science 205: 179–187.

[nph16897-bib-0003] Arshad MS , Farooq M , Asch F , Krishna JSV , Prasad PVV , Siddique KHM . 2017 Thermal stress impacts reproductive development and grain yield in rice. Plant Physiology and Biochemistry 115: 57–72. 28324683 10.1016/j.plaphy.2017.03.011

[nph16897-bib-0004] Bahuguna RN , Solis CA , Shi W , Jagadish KSV . 2017 Post‐flowering night respiration and altered sink activity account for high night temperature‐induced grain yield and quality loss in rice (*Oryza sativa* L.). Physiologia Plantarum 159: 59–73. 27513992 10.1111/ppl.12485

[nph16897-bib-0005] Bai X , Zhao H , Huang Y , Xie W , Han Z , Zhang B , Guo Z , Yang L , Dong H , Xue W *et al* 2016 Genome‐wide association analysis reveals different genetic control in panicle architecture between *Indica* and *Japonica* rice. Plant Genome 9: 2. 10.3835/plantgenome2015.11.0115 27898816

[nph16897-bib-0006] Bailey‐Serres J , Parker JE , Ainsworth EA , Oldroyd GED , Schroeder JI . 2019 Genetic strategies for improving crop yields. Nature 575: 109–118. 31695205 10.1038/s41586-019-1679-0 PMC7024682

[nph16897-bib-0007] Batista RA , Figueiredo DD , Santos‐González J , Köhler C . 2019 Auxin regulates endosperm cellularization in Arabidopsis. Genes and Development 33: 466–476. 30819818 10.1101/gad.316554.118 PMC6446538

[nph16897-bib-0008] Berendzen K , Searle I , Ravenscroft D , Koncz C , Batschauer A , Coupland G , Somssich IE , Ülker B . 2005 A rapid and versatile combined DNA/RNA extraction protocol and its application to the analysis of a novel DNA marker set polymorphic between Arabidopsis thaliana ecotypes Col‐0 and Landsberg erecta. Plant Methods 1: 4. 16270938 10.1186/1746-4811-1-4 PMC1277017

[nph16897-bib-0009] Bokszczanin KL , Fragkostefanakis S . 2013 Perspectives on deciphering mechanisms underlying plant heat stress response and thermotolerance. Frontiers in Plant Science 4: 1–20. 23986766 10.3389/fpls.2013.00315 PMC3750488

[nph16897-bib-0010] Che R , Tong H , Shi B , Liu Y , Fang S , Liu D , Xiao Y , Hu B , Liu L , Wang H *et al* 2015 Control of grain size and rice yield by *GL2*‐mediated brassinosteroid responses. Nature Plants 1: 15195. 10.1038/nplants.2015.195 27250747

[nph16897-bib-0011] Chen C , Begcy K , Liu K , Folsom JJ , Wang Z , Zhang C , Walia H . 2016 Heat stress yields a unique MADS box transcription factor in determining seed size and thermal sensitivity. Plant Physiology 171: 606–622. 26936896 10.1104/pp.15.01992 PMC4854699

[nph16897-bib-0012] Cheng W , Sakai H , Yagi K , Hasegawa T . 2009 Interactions of elevated CO_2_ and night temperature on rice growth and yield. Agricultural and Forest Meteorology 149: 51–58.

[nph16897-bib-0013] Coast O , Ellis RH , Murdoch AJ , Quiñones C , Jagadish KSV . 2015 High night temperature induces contrasting responses for spikelet fertility, spikelet tissue temperature, flowering characteristics and grain quality in rice. Functional Plant Biology 42: 149. 32480661 10.1071/FP14104

[nph16897-bib-0014] Covarrubias‐Pazaran G . 2016 Genome‐assisted prediction of quantitative traits using the R Package sommer. PLoS ONE 11: e0156744. 27271781 10.1371/journal.pone.0156744 PMC4894563

[nph16897-bib-0015] Dhatt BK , Abshire N , Paul P , Hasanthika K , Sandhu J , Zhang Q , Obata T , Walia H . 2019 Metabolic dynamics of developing rice seeds under high night‐time temperature stress. Frontiers in Plant Science 10: 1443. 31781147 10.3389/fpls.2019.01443 PMC6857699

[nph16897-bib-0016] Duan P , Ni S , Wang J , Zhang B , Xu R , Wang Y , Chen H , Zhu X , Li Y . 2015 Regulation of *OsGRF4* by OsmiR396 controls grain size and yield in rice. Nature Plants 1: 15203. 10.1038/nplants.2015.203 27250749

[nph16897-bib-0017] Duan P , Xu J , Zeng D , Zhang B , Geng M , Zhang G , Huang K , Huang L , Xu R , Ge S *et al* 2017 Natural variation in the promoter of GSE5 contributes to grain size diversity in rice. Molecular Plant 10: 685–694. 28366824 10.1016/j.molp.2017.03.009

[nph16897-bib-0018] Easterling DR , Horton B , Jones PD , Peterson TC , Karl TR , Parker DE , Salinger MJ , Razuvayev V , Plummer N , Jamason P *et al* 1997 Maximum and minimum temperature trends for the globe. Science 277: 364–367.

[nph16897-bib-0019] Eizenga GC , Ali ML , Bryant RJ , Yeater KM , McClung AM , McCouch SR . 2014 Registration of the rice diversity panel 1 for genomewide association studies. Journal of Plant Registrations 8: 109–116.

[nph16897-bib-0020] Endelman JB . 2011 Ridge regression and other kernels for genomic selection with R Package rrBLUP. Plant Genome Journal 4: 250.

[nph16897-bib-0021] Fan C , Xing Y , Mao H , Lu T , Han B , Xu C , Li X , Zhang Q . 2006 GS3, a major QTL for grain length and weight and minor QTL for grain width and thickness in rice, encodes a putative transmembrane protein. Theoretical and Applied Genetics 112: 1164–1171. 16453132 10.1007/s00122-006-0218-1

[nph16897-bib-0022] Fang CY , Zhang H , Wan J , Wu YY , Li K , Jin C , Chen W , Wang SC , Wang WS , Zhang HW *et al* 2016 Control of leaf senescence by an MeOH‐Jasmonates cascade that is epigenetically regulated by OsSRT1 in rice. Molecular Plant 9: 1366–1378. 27477683 10.1016/j.molp.2016.07.007

[nph16897-bib-0023] Figueiredo DD , Batista RA , Roszak PJ , Hennig L , Köhler C . 2016 Auxin production in the endosperm drives seed coat development in Arabidopsis. eLife 5: 1–23. 10.7554/eLife.20542 PMC5135394 27848912

[nph16897-bib-0024] Fitzgerald MA , McCouch SR , Hall RD . 2009 Not just a grain of rice: the quest for quality. Trends in Plant Science 14: 133–139. 19230745 10.1016/j.tplants.2008.12.004

[nph16897-bib-0025] Foley JA , Ramankutty N , Brauman KA , Cassidy ES , Gerber JS , Johnston M , Mueller ND , O’Connell C , Ray DK , West PC *et al* 2011 Solutions for a cultivated planet. Nature 478: 337–342. 21993620 10.1038/nature10452

[nph16897-bib-0026] Folsom JJ , Begcy K , Hao X , Wang D , Walia H . 2014 Rice fertilization‐Independent Endosperm1 regulates seed size under heat stress by controlling early endosperm development. Plant Physiology 165: 238–248. 24590858 10.1104/pp.113.232413 PMC4012583

[nph16897-bib-0027] Fragkostefanakis S , Mesihovic A , Simm S , Paupière MJ , Hu Y , Paul P , Mishra SK , Tschiersch B , Theres K , Bovy A *et al* 2016 HsfA2 controls the activity of developmentally and stress‐regulated heat stress protection mechanisms in tomato male reproductive tissues. Plant Physiology 170: 2461–2477. 26917685 10.1104/pp.15.01913 PMC4825147

[nph16897-bib-0028] Fragkostefanakis S , Simm S , Paul P , Bublak D , Scharf K‐D , Schleiff E . 2015 Chaperone network composition in Solanum lycopersicum explored by transcriptome profiling and microarray meta‐analysis. Plant, Cell & Environment 38: 693–709. 10.1111/pce.12426 25124075

[nph16897-bib-0029] Gehring M . 2013 Genomic imprinting: insights from plants. Annual Review of Genetics 47: 187–208. 10.1146/annurev-genet-110711-155527 24016190

[nph16897-bib-0030] Hasanuzzaman M , Nahar K , Alam M , Roychowdhury R , Fujita M . 2013 Physiological, biochemical, and molecular mechanisms of heat stress tolerance in plants. International Journal of Molecular Sciences 14: 9643–9684. 23644891 10.3390/ijms14059643 PMC3676804

[nph16897-bib-0031] Hong Z , Huang R , Jiang L , Zheng J , Wang T , Wang H , Huang Y , Hong Z . 2015 Genetic bases of rice grain shape: so many genes, so little known. Trends in Plant Science 18: 218–226. 10.1016/j.tplants.2012.11.001 23218902

[nph16897-bib-0032] Hu J , Wang Y , Fang Y , Zeng L , Xu J , Yu H , Shi Z , Pan J , Zhang D , Kang S *et al* 2015 A rare allele of GS2 enhances grain size and grain yield in rice. Molecular Plant 8: 1455–1465. 26187814 10.1016/j.molp.2015.07.002

[nph16897-bib-0033] Huang X , Lu Z , Wang X , Ouyang Y , Chen W , Xie K , Wang D , Luo M , Luo J , Yao J . 2016 Imprinted gene *OsFIE1* modulates rice seed development by influencing nutrient metabolism and modifying genome H3K27me3. The Plant Journal 87: 305–317. 27133784 10.1111/tpj.13202

[nph16897-bib-0034] Huang X , Wei X , Sang T , Zhao Q , Feng Q , Zhao Y , Li C , Zhu C , Lu T , Zhang Z *et al* 2010 Genome‐wide asociation studies of 14 agronomic traits in rice landraces. Nature Genetics 42: 961–967. 20972439 10.1038/ng.695

[nph16897-bib-0035] Ishimaru T , Horigane AK , Ida M , Iwasawa N , San‐oh YA , Nakazono M , Nishizawa NK , Masumura T , Kondo M , Yoshida M . 2009 Formation of grain chalkiness and changes in water distribution in developing rice caryopses grown under high‐temperature stress. Journal of Cereal Science 50: 166–174.

[nph16897-bib-0036] Jagadish SVK , Murt MVR , Quick WP . 2015 Rice responses to rising temperatures ‐ challenges, perspectives and future directions. Plant, Cell & Environment 38: 1686–1698. 10.1111/pce.12430 25142172

[nph16897-bib-0037] Jain M , Nijhawan A , Tyagi AK , Khurana JP . 2006 Validation of housekeeping genes as internal control for studying gene expression in rice by quantitative real‐time PCR. Biochemical and Biophysical Research Communications 345: 646–651. 16690022 10.1016/j.bbrc.2006.04.140

[nph16897-bib-0038] Kadan RS , Bryant RJ , Miller JA . 2008 Effects of milling on functional properties of rice flour. Journal of Food Science 73: E151–E154. 18460123 10.1111/j.1750-3841.2008.00720.x

[nph16897-bib-0039] Karl TR , Jones PD , Knight RW , Kukla G , Plummer N , Karl TR , Jones PD , Knight RW , Kukla G , Plummer N *et al* 1993 Asymmetric trends of daily maximum and minimum temperature. Bulletin of the American Meteorological Society 74: 1007–1024.

[nph16897-bib-0040] Khush GS . 2005 What it will take to feed 5.0 billion rice consumers in 2030. Plant Molecular Biology 59: 1–6. 16217597 10.1007/s11103-005-2159-5

[nph16897-bib-0041] Khush GS , Jena KK . 2009 Current status and future prospects for research on blast resistance in rice (*Oryza sativa* L.) In: Advances in genetics, genomics and control of rice blast disease. Dordrecht, the Netherlands: Springer, 1–10.

[nph16897-bib-0042] Kumar A , Kumar S , Prasad M , Thakur JK . 2019 Designing of a mini‐core that effectively represents 3004 diverse accessions of rice. bioRxiv: 762070. doi: 10.1101/762070.

[nph16897-bib-0043] Lei Y , Lu L , Liu H‐Y , Li S , Xing F , Chen L‐L . 2014 CRISPR‐P: a web tool for synthetic single‐guide RNA design of CRISPR‐system in plants. Molecular Plant 7: 1494–1496. 24719468 10.1093/mp/ssu044

[nph16897-bib-0044] Li N , Xu R , Duan P , Li Y . 2018 Control of grain size in rice. Plant Reproduction 31: 237–251. 29523952 10.1007/s00497-018-0333-6

[nph16897-bib-0045] Li N , Xu R , Li Y . 2019 Molecular networks of seed size control in plants. Annual Review of Plant Biology 70: 435–463. 10.1146/annurev-arplant-050718-095851 30795704

[nph16897-bib-0046] Li S , Gao F , Xie K , Zeng X , Cao Y , Zeng J , He Z , Ren Y , Li W , Deng Q *et al* 2016 The OsmiR396c‐OsGRF4‐OsGIF1 regulatory module determines grain size and yield in rice. Plant Biotechnology Journal 14: 2134–2146. 27107174 10.1111/pbi.12569 PMC5095787

[nph16897-bib-0047] Liakat Ali M , McClung AM , Jia MH , Kimball JA , McCouch SR , Georgia CE . 2011 A rice diversity panel evaluated for genetic and agro‐morphological diversity between subpopulations and its geographic distribution. Crop Science 51: 2021–2035.

[nph16897-bib-0048] Lisle AJ , Martin M , Fitzgerald MA . 2000 Chalky and translucent rice grains differ in starch composition and structure and cooking properties. Cereal Chemistry Journal 77: 627–632.

[nph16897-bib-0049] Liu J , Chen J , Zheng X , Wu F , Lin Q , Heng Y , Tian P , Cheng Z , Yu X , Zhou K *et al* 2017 *GW5* acts in the brassinosteroid signalling pathway to regulate grain width and weight in rice. Nature Plants 3: 17043. 28394310 10.1038/nplants.2017.43

[nph16897-bib-0050] Livak KJ , Schmittgen TD . 2001 Analysis of relative gene expression data using real‐time quantitative PCR and the 2^−ΔΔCT^ method. Methods 25: 402–408. 11846609 10.1006/meth.2001.1262

[nph16897-bib-0051] Lizana XC , Riegel R , Gomez LD , Herrera J , Isla A , McQueen‐Mason SJ , Calderini DF . 2010 Expansins expression is associated with grain size dynamics in wheat (*Triticum aestivum* L.). Journal of Experimental Botany 61: 1147–1157. 20080826 10.1093/jxb/erp380 PMC2826655

[nph16897-bib-0052] Lobell DB . 2007 Changes in diurnal temperature range and national cereal yields. Agricultural and Forest Meteorology 145: 229–238.

[nph16897-bib-0053] Lowder LG , Zhang D , Baltes NJ , Paul JW , Tang X , Zheng X , Voytas DF , Hsieh T‐F , Zhang Y , Qi Y . 2015 A CRISPR/Cas9 toolbox for multiplexed plant genome editing and transcriptional regulation. Plant Physiology 169: 971–985. 26297141 10.1104/pp.15.00636 PMC4587453

[nph16897-bib-0054] Luo M , Platten D , Chaudhury A , Peacock WJ , Dennis ES . 2009 Expression, imprinting, and evolution of rice homologs of the polycomb group genes. Molecular Plant 2: 711–723. 19825651 10.1093/mp/ssp036

[nph16897-bib-0055] Lyman NB , Jagadish KSV , Nalley LL , Dixon BL , Siebenmorgen T . 2013 Neglecting rice milling yield and quality underestimates economic losses from high‐temperature stress. PLoS ONE 8: e72157. 23991056 10.1371/journal.pone.0072157 PMC3750041

[nph16897-bib-0056] Mao H , Sun S , Yao J , Wang C , Yu S , Xu C , Li X , Zhang Q . 2010 Linking differential domain functions of the GS3 protein to natural variation of grain size in rice. Proceedings of the National Academy of Sciences, USA 107: 19579–19584. 10.1073/pnas.1014419107 PMC2984220 20974950

[nph16897-bib-0057] McCouch SR , Wright MH , Tung CW , Maron LG , McNally KL , Fitzgerald M , Singh N , DeClerck G , Agosto‐Perez F , Korniliev P *et al* 2016 Open access resources for genome‐wide association mapping in rice. Nature Communications 7: 10532. 10.1038/ncomms10532 PMC4742900 26842267

[nph16897-bib-0058] Mohammed AR , Cothren JT , Tarpley L . 2013 High night temperature and abscisic acid affect rice productivity through altered photosynthesis, respiration and spikelet fertility. Crop Science 53: 2603–2612.

[nph16897-bib-0059] Morita S , Yonemaru J‐I , Takanashi J‐I . 2005 Grain growth and endosperm cell size under high night temperatures in rice (*Oryza sativa* L.). Annals of Botany 95: 695–701. 15655104 10.1093/aob/mci071 PMC4246861

[nph16897-bib-0060] Muthayya S , Sugimoto JD , Montgomery S , Maberly GF . 2014 An overview of global rice production, supply, trade, and consumption. Annals of the New York Academy of Sciences 1324: 7–14. 25224455 10.1111/nyas.12540

[nph16897-bib-0061] Olsen O‐A . 2004 Nuclear endosperm development in cereals and *Arabidopsis thaliana* . The Plant Cell 16: S214–S227. 15010513 10.1105/tpc.017111 PMC2643391

[nph16897-bib-0062] Padma Kumari B , Londhe AL , Daniel S , Jadhav DB . 2007 Observational evidence of solar dimming: offsetting surface warming over India. Geophysical Research Letters 34: L21810.

[nph16897-bib-0063] Paul P , Dhatt BK , Miller M , Folsom JJ , Wang Z , Krassovskaya I , Liu K , Sandhu J , Yu H , Zhang C *et al* 2020a *MADS78* and *MADS79* are essential regulators of early seed development in rice. Plant Physiology 182: 933–948. 31818903 10.1104/pp.19.00917 PMC6997703

[nph16897-bib-0064] Paul P , Dhatt BK , Sandhu J , Hussain W , Irvin L , Morota G , Staswick P , Walia H . 2020b Divergent phenotypic response of rice accessions to transient heat stress during early seed development. Plant Direct 4: 1–13. 10.1002/pld3.196 PMC6955394 31956854

[nph16897-bib-0065] Peng S , Huang J , Sheehy JE , Laza RC , Visperas RM , Zhong X , Centeno GS , Khush GS , Cassman KG . 2004 Rice yields decline with higher night temperature from global warming. Proceedings of the National Academy of Sciences, USA 101: 9971–9975. 10.1073/pnas.0403720101 PMC454199 15226500

[nph16897-bib-0066] Peraudeau S , Lafarge T , Roques S , Quiñones CO , Clement‐Vidal A , Ouwerkerk PBF , Van Rie J , Fabre D , Jagadish KSV , Dingkuhn M . 2015 Effect of carbohydrates and night temperature on night respiration in rice. Journal of Experimental Botany 66: 3931–3944. 25954047 10.1093/jxb/erv193

[nph16897-bib-0067] Pérez P , De Los Campos G . 2014 Genome‐wide regression and prediction with the BGLR statistical package. Genetics 198: 483–495. 25009151 10.1534/genetics.114.164442 PMC4196607

[nph16897-bib-0068] Philipp N , Weichert H , Bohra U , Weschke W , Schulthess AW , Weber H . 2018 Grain number and grain yield distribution along the spike remain stable despite breeding for high yield in winter wheat. PLoS ONE 13: e0205452. 30304020 10.1371/journal.pone.0205452 PMC6179273

[nph16897-bib-0069] Pielot R , Kohl S , Manz B , Rutten T , Weier D , Tarkowská D , Rolčík J , Strnad M , Volke F , Weber H *et al* 2015 Hormone‐mediated growth dynamics of the barley pericarp as revealed by magnetic resonance imaging and transcript profiling. Journal of Experimental Botany 66: 6927–6943. 26276866 10.1093/jxb/erv397 PMC4623697

[nph16897-bib-0070] Pingali PL . 2012 Green revolution: impacts, limits, and the path ahead. Proceedings of the National Academy of Sciences, USA 109: 12302–12308. 10.1073/pnas.0912953109 PMC3411969 22826253

[nph16897-bib-0071] Pires ND . 2014 Seed evolution: parental conflicts in a multi‐generational household. Biomolecular Concepts 5: 71–86. 25372743 10.1515/bmc-2013-0034

[nph16897-bib-0072] Porter JR , Gawith M . 1999 Temperatures and the growth and development of wheat: a review. European Journal of Agronomy 10: 23–36.

[nph16897-bib-0073] Prasad PVV , Boote KJ , Allen LH , Sheehy JE , Thomas JMG . 2006 Species, ecotype and cultivar differences in spikelet fertility and harvest index of rice in response to high temperature stress. Field Crops Research 95: 398–411.

[nph16897-bib-0074] R Core Team . 2019 R: a language and environment for statistical computing, Version 3.0.2. Vienna, Austria: R foundation for statistical computing.

[nph16897-bib-0075] Röth S , Paul P , Fragkostefanakis S . 2016 Plant heat stress response and thermotolerance In: Jaiwal P, Singh R, Dhankher O, eds. Genetic manipulation in plants for mitigation of climate change. New Delhi, India: Springer, 15–41.

[nph16897-bib-0076] Sakamoto T , Matsuoka M . 2008 Identifying and exploiting grain yield genes in rice. Current opinion in plant biology 11: 209–214. 18343712 10.1016/j.pbi.2008.01.009

[nph16897-bib-0077] Sandhu J , Zhu F , Paul P , Gao T , Dhatt BK , Ge Y , Staswick P , Yu H , Walia H . 2019 PI‐Plat: A high‐resolution image‐based 3D reconstruction method to estimate growth dynamics of rice inflorescence traits. Plant Methods 15: 162. 31889986 10.1186/s13007-019-0545-2 PMC6933716

[nph16897-bib-0078] Shen X , De Jonge J , Forsberg SKG , Pettersson ME , Sheng Z , Hennig L , Carlborg Ö . 2014 Natural *CMT2* Variation Is Associated With Genome‐Wide Methylation Changes and Temperature Seasonality. PLoS Genetics 10: e1004842. 25503602 10.1371/journal.pgen.1004842 PMC4263395

[nph16897-bib-0079] Shi W , Muthurajan R , Rahman H , Selvam J , Peng S , Zou Y , Jagadish KSV . 2013 Source‐sink dynamics and proteomic reprogramming under elevated night temperature and their impact on rice yield and grain quality. New Phytologist 197: 825–837. 10.1111/nph.12088 23252708

[nph16897-bib-0080] Shindo C , Lister C , Crevillen P , Nordborg M , Dean C . 2006 Variation in the epigenetic silencing of FLC contributes to natural variation in Arabidopsis vernalization response. Genes & Development 20: 3079–3083. 17114581 10.1101/gad.405306 PMC1635143

[nph16897-bib-0081] Shomura A , Izawa T , Ebana K , Ebitani T , Kanegae H , Konishi S , Yano M . 2008 Deletion in a gene associated with grain size increased yields during rice domestication. Nature Genetics 40: 1023–1028. 18604208 10.1038/ng.169

[nph16897-bib-0082] Si L , Chen J , Huang X , Gong H , Luo J , Hou Q , Zhou T , Lu T , Zhu J , Shangguan Y *et al* 2016 *OsSPL13* controls grain size in cultivated rice. Nature Genetics 48: 447–456. 26950093 10.1038/ng.3518

[nph16897-bib-0083] Song XJ , Kuroha T , Ayano M , Furuta T , Nagai K , Komeda N , Segami S , Miura K , Ogawa D , Kamura T *et al* 2015 Rare allele of a previously unidentified histone H4 acetyltransferase enhances grain weight, yield, and plant biomass in rice. Proceedings of the National Academy of Sciences, USA 112: 76–81. 10.1073/pnas.1421127112 PMC4291654 25535376

[nph16897-bib-0084] Sreenivasulu N , Butardo VM , Misra G , Cuevas RP , Anacleto R , Kishor PBK . 2015 Designing climate‐resilient rice with ideal grain quality suited for high‐temperature stress. Journal of Experimental Botany 66: 1737–1748. 25662847 10.1093/jxb/eru544 PMC4669556

[nph16897-bib-0085] Sun LP , Zhang YX , Zhang PP , Yang ZF , Zhou XX , Xuan DD , Rahman HH , Li ZH , Wu WX , Zhan XD *et al* 2017 Morphogenesis and Gene Mapping of *deformed interior floral organ 1* (*difo1*), a Novel Mutant Associated with Floral Organ Development in Rice. Plant Molecular Biology Reporter 35: 130–144.

[nph16897-bib-0086] Sun P , Zhang W , Wang Y , He Q , Shu F , Liu H , Wang J , Wang J , Yuan L , Deng H . 2016 *OsGRF4* controls grain shape, panicle length and seed shattering in rice. Journal of Integrative Plant Biology 58: 836–847. 26936408 10.1111/jipb.12473 PMC5089622

[nph16897-bib-0087] Sun X , Ren G , You Q , Ren Y , Xu W , Xue X , Zhan Y , Zhang S , Zhang P . 2019 Global diurnal temperature range (DTR) changes since 1901. Climate Dynamics 52: 3343–3356.

[nph16897-bib-0088] Sweeney MT , Thomson MJ , Pfeil BE , McCouch S . 2006 Caught red‐handed: *Rc* encodes a basic helix‐loop‐helix protein conditioning red pericarp in rice. The Plant Cell 18: 283–294. 16399804 10.1105/tpc.105.038430 PMC1356539

[nph16897-bib-0089] Tashiro T , Wardlaw I . 1991 The effect of high temperature on kernel dimensions and the type and occurrence of kernel damage in rice. Australian Journal of Agricultural Research 42: 485.

[nph16897-bib-0090] Thorne PW , Donat MG , Dunn RJH , Williams CN , Alexander LV , Caesar J , Durre I , Harris I , Hausfather Z , Jones PD *et al* 2016 Reassessing changes in diurnal temperature range: Intercomparison and evaluation of existing global data set estimates. Journal of Geophysical Research: Atmospheres 121: 5138–5158.

[nph16897-bib-0091] Tsutsui K , Kaneko K , Hanashiro I , Nishinari K , Toshiaki M . 2013 Characteristics of opaque and translucent parts of high temperature stressed grains of rice. Journal of Applied Glycoscience 60: 61–67.

[nph16897-bib-0092] Turner SD . 2014 qqman: an R package for visualizing GWAS results using Q‐Q and manhattan plots. bioRxiv: 005165. doi: 10.1101/005165.

[nph16897-bib-0093] Vose RS , Easterling DR , Gleason B . 2005 Maximum and minimum temperature trends for the globe: an update through 2004. Geophysical Research Letters 32: L23822.

[nph16897-bib-0112] Wada H , Hatakeyama Y , Onda Y , Nonami H , Nakashima T , Erra‐Balsells R , Morita S , Hiraoka K , Tanaka F , Nakano H. 2019 Multiple strategies for heat adaptation to prevent chalkiness in the rice endosperm. Journal of Experimental Botany 70: 1299–1311. 30508115 10.1093/jxb/ery427 PMC6382329

[nph16897-bib-0094] Wang S , Wu K , Yuan Q , Liu X , Liu Z , Lin X , Zeng R , Zhu H , Dong G , Qian Q *et al* 2012 Control of grain size, shape and quality by *OsSPL16* in rice. Nature Genetics 44: 950–954. 22729225 10.1038/ng.2327

[nph16897-bib-0095] Wang Y , Ren Y , Liu X , Jiang L , Chen L , Han X , Jin M , Liu S , Liu F , Lv J *et al* 2010 OsRab5a regulates endomembrane organization and storage protein trafficking in rice endosperm cells. The Plant Journal 64: 812–824. 21105928 10.1111/j.1365-313X.2010.04370.x

[nph16897-bib-0096] Welch JR , Vincent JR , Auffhammer M , Moya PF , Dobermann A , Dawe D . 2010 Rice yields in tropical/subtropical Asia exhibit large but opposing sensitivities to minimum and maximum temperatures. Proceedings of the National Academy of Sciences, USA 107: 14562–14567. 10.1073/pnas.1001222107 PMC2930450 20696908

[nph16897-bib-0097] Weng J , Gu S , Wan X , Gao H , Guo T , Su N , Lei C , Zhang X , Cheng Z , Guo X *et al* 2008 Isolation and initial characterization of GW5, a major QTL associated with rice grain width and weight. Cell Research 18: 1199–1209. 19015668 10.1038/cr.2008.307

[nph16897-bib-0098] Wik M , Pingali P , Broca S . 2008 Background paper for the world development report 2008: global agricultural performance: past trends and future prospects. Washington, DC, USA: World Bank.

[nph16897-bib-0099] Wu W , Liu X , Wang M , Meyer RS , Luo X , Ndjiondjop MN , Tan L , Zhang J , Wu J , Cai H *et al* 2017 A single‐nucleotide polymorphism causes smaller grain size and loss of seed shattering during African rice domestication. Nature Plants 3: 17064. 28481332 10.1038/nplants.2017.64

[nph16897-bib-0100] Xing Y , Zhang Q . 2010 Genetic and molecular bases of rice yield. Annual Review of Plant Biology 61: 421–442. 10.1146/annurev-arplant-042809-112209 20192739

[nph16897-bib-0101] Yang J , Zeng J , Goddard ME , Wray NR , Visscher PM . 2017 Concepts, estimation and interpretation of SNP‐based heritability. Nature Genetics 49: 1304–1310. 28854176 10.1038/ng.3941

[nph16897-bib-0102] Zhang L , Cheng Z , Qin R , Qiu Y , Wang J‐L , Cui X , Gu L , Zhang X , Guo X , Wang D *et al* 2012 Identification and characterization of an Epi‐allele of *FIE1* reveals a regulatory linkage between two epigenetic marks in rice. The Plant Cell 24: 4407–4421. 23150632 10.1105/tpc.112.102269 PMC3531842

[nph16897-bib-0103] Zhao C , Liu B , Piao S , Wang X , Lobell DB , Huang Y , Huang M , Yao Y , Bassu S , Ciais P *et al* 2017 Temperature increase reduces global yields of major crops in four independent estimates. Proceedings of the National Academy of Sciences, USA 114: 9326–9331. 10.1073/pnas.1701762114 PMC5584412 28811375

[nph16897-bib-0104] Zhao D‐S , Li Q‐F , Zhang C‐Q , Zhang C , Yang Q‐Q , Pan L‐X , Ren X‐Y , Lu J , Gu M‐H , Liu Q‐Q . 2018 GS9 acts as a transcriptional activator to regulate rice grain shape and appearance quality. Nature Communications 9: 1240. 10.1038/s41467-018-03616-y PMC5869696 29588443

[nph16897-bib-0105] Zhao K , Tung C‐W , Eizenga GC , Wright MH , Liakat Ali M , Price AH , Norton GJ , Rafi qul Islam M , Reynolds A , Mezey J , *et al* 2011 Genome‐wide association mapping reveals a rich genetic architecture of complex traits in Oryza sativa. Nature Communications 2: 467. 10.1038/ncomms1467 PMC3195253 21915109

[nph16897-bib-0106] Zheng X , Levine D , Shen J , Gogarten SM , Laurie C , Weir BS . 2012 A high‐performance computing toolset for relatedness and principal component analysis of SNP data. Bioinformatics 28: 3326–3328. 23060615 10.1093/bioinformatics/bts606 PMC3519454

[nph16897-bib-0107] Zhou L , Dickinson RE , Tian Y , Fang J , Li Q , Kaufmann RK , Tucker CJ , Myneni RB . 2004 Evidence for a significant urbanization effect on climate in China. PNAS 101: 9540–9544. 15205480 10.1073/pnas.0400357101 PMC470710

[nph16897-bib-0108] Zhu F , Paul P , Hussain W , Wallman K , Dhatt BK , Irvin L , Morota G , Yu H , Walia H . 2020 SeedExtractor: an open‐source GUI for seed image analysis. bioRxiv: 176230. doi: 10.1101/2020.06.28.176230. PMC7882627 33597957

[nph16897-bib-0109] Zhu Y , Lin Y , Chen S , Liu H , Chen Z , Fan M , Hu T , Mei F , Chen J , Chen L *et al* 2019 CRISPR/Cas9‐mediated functional recovery of the recessive *rc* allele to develop red rice. Plant Biotechnology Journal 17: 2096–2105. 31002444 10.1111/pbi.13125 PMC6790373

[nph16897-bib-0110] Zinn KE , Tunc‐Ozdemir M , Harper JF . 2010 Temperature stress and plant sexual reproduction: uncovering the weakest links. Journal of Experimental Botany 61: 1959–1968. 20351019 10.1093/jxb/erq053 PMC2917059

[nph16897-bib-0111] Ziska LH , Manalo PA . 1996 Increasing night temperature can reduce seed set and potential yield of tropical rice. Australian Journal of Plant Physiology 23: 791–794.

