# Supplementary material for: Allelic variation in rice Fertilization Independent Endosperm 1 contributes to grain width under high night temperature stress
Source: New Phytol. 2020 Sep 23;229(1):335–50. doi: 10.1111/nph.16897 (PMC7756756; doi:10.1111/nph.16897)
Supplement: Supplementary file 1 — Fig. S1 Principal component analysis (PCA) showing the population structure of the rice diversity association panel (RDP1) used in this study. Fig. S2 Phenotypic distribution of rice mature grain length and width under control and high night temperature (HNT) stress. Fig. S3 Rice subpopulation‐level phenotypic distribution of mature grain length and width under control and high night temperature (HNT) stress. Fig. S4 Q‐Q plots of –log10(P) values obtained from the linear mixed model for mature grain length and width under control and high night temperature (HNT) stress in rice. Fig. S5 Relative transcript abundance of Fie1 in wild‐type (WT) developing seeds (4, 7, and 10 d after fertilization; DAF) under control conditions in rice. Fig. S6 DNA methylation analysis. Fig. S7 Relative transcript abundance of Fie1 neighboring genes (two upstream and two downstream) in developing seeds in rice. Fig. S8 The mutants used in the study: Fie1 overexpression (a; fie1OE10 and fie1OE11) and knockouts (b; fie1CR2 and fie1CR3) in rice. Fig. S9 Representative images of wild‐type (WT), knockout (fie1CR2 and fie1CR3), and overexpression (fie1OE10 and fie1OE11) mutants at day 65 in rice. Fig. S10 RT‐qPCR analysis of rice grain size‐related genes. Fig. S11 RT‐qPCR analysis for selected set of rice starch biosynthesis genes in the mutants. Fig. S12 Cross‐sections of mature grains from four major (M1–M4) and four minor (m1–m4) allelic accessions under HNT observed via scanning electron microscopy. [file NPH-229-335-s001.pdf]

New Phytologist Supporting Information

Article title: Allelic variation in rice *Fertilization Independent Endosperm 1* contributes to grain width under high night temperature stress

Authors: Balpreet K Dhatt, Puneet Paul, Jaspreet Sandhu, Waseem Hussain, Larissa Irvin, Feiyu Zhu, Maria Arlene Adviento-Borbe, Argelia Lorence, Paul Staswick, Hongfeng Yu, Gota Morota and Harkamal Walia

Article acceptance date: 9 August 2020

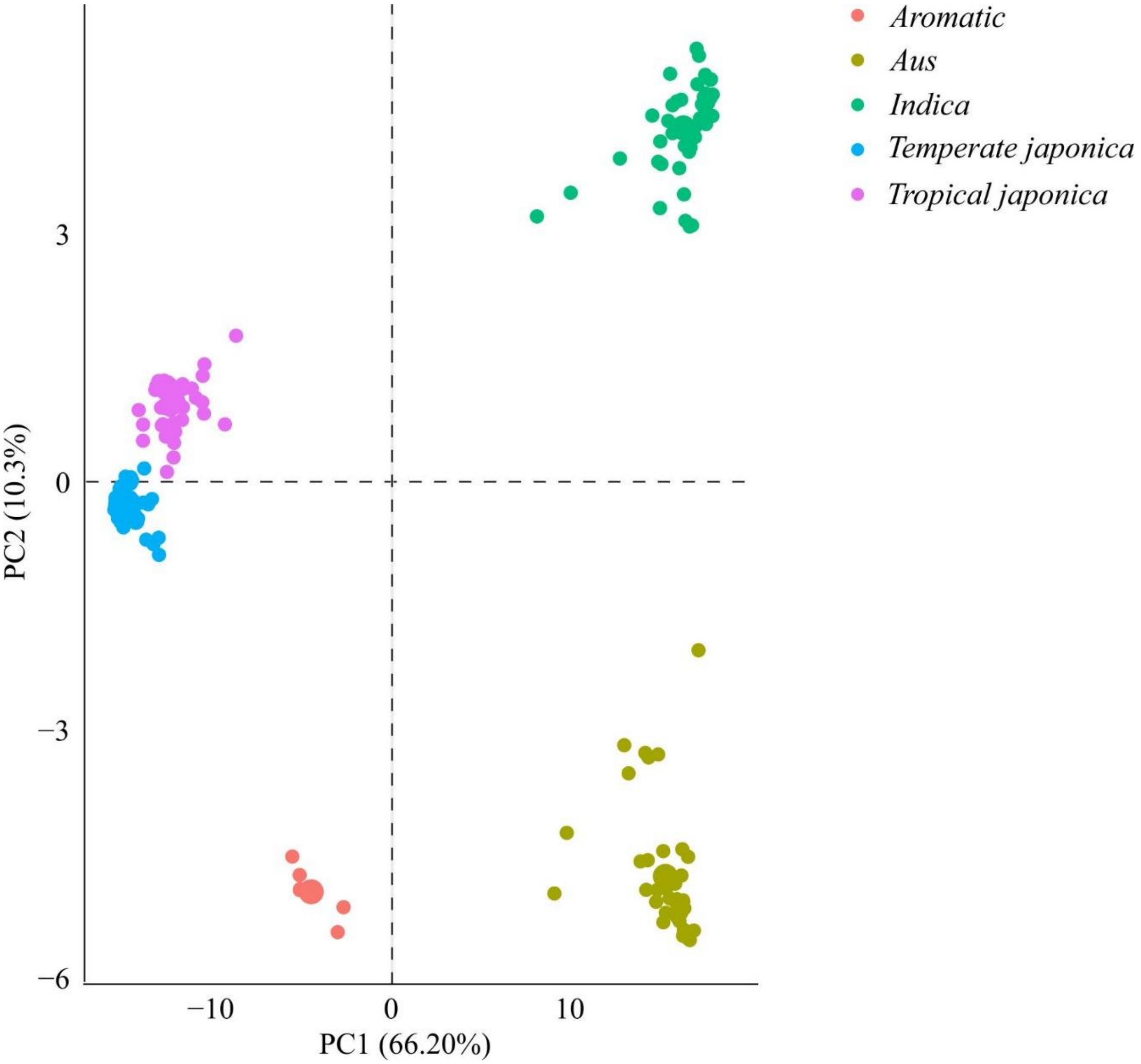

Figure S1. Principal component analysis (PCA) showing the population structure of Rice diversity association panel (RDP1) used in this study. The rice diversity panel is divided into five different sub-populations represented by different colors in the figure. PCA was performed on the genomic relationship matrix derived from 700k marker data and the first two components are plotted to show the population structure of rice diversity panel.

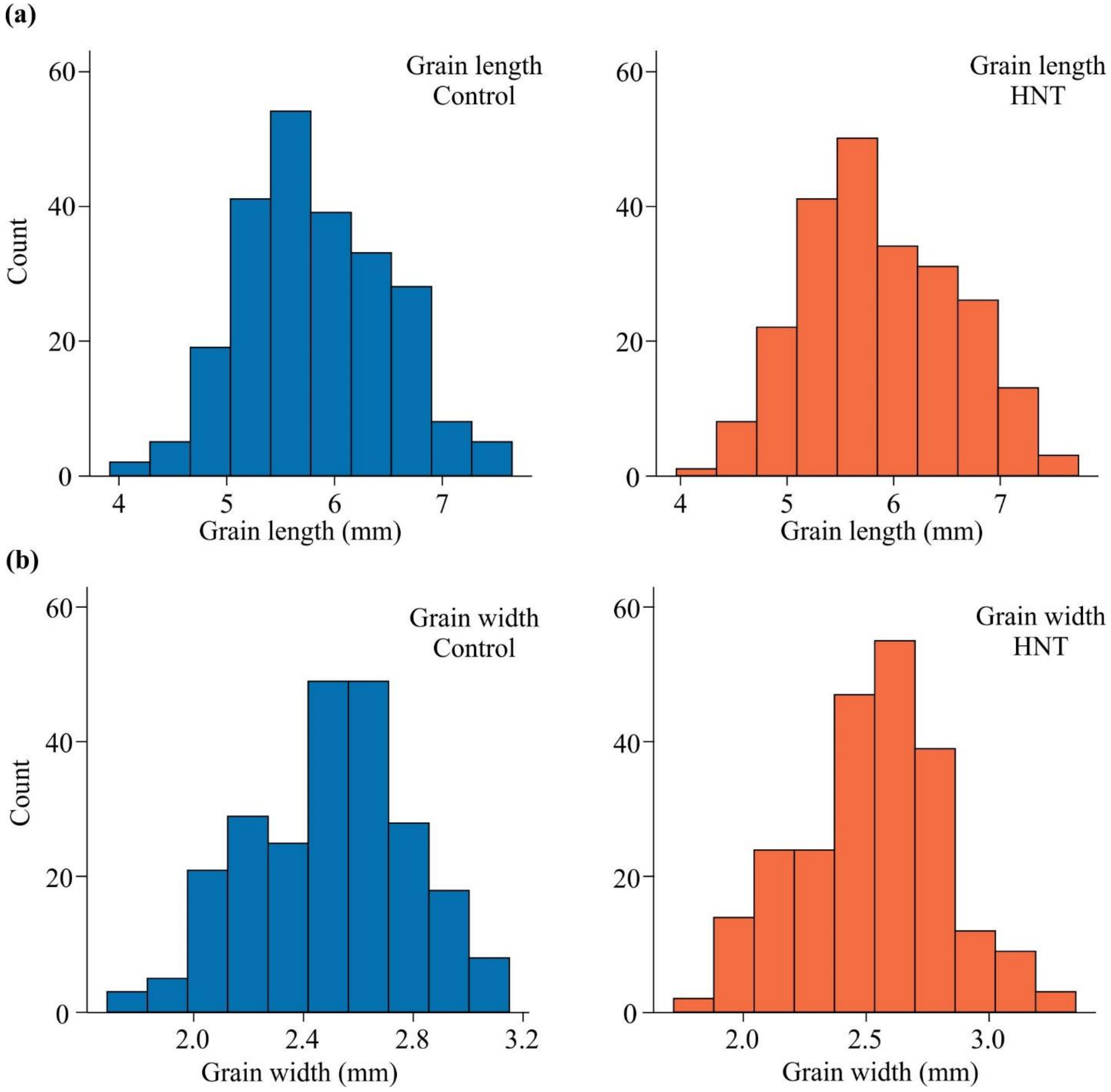

Figure S2: Phenotypic distribution of mature grain length (a) and width (b) under control and high night temperature (HNT) stress.

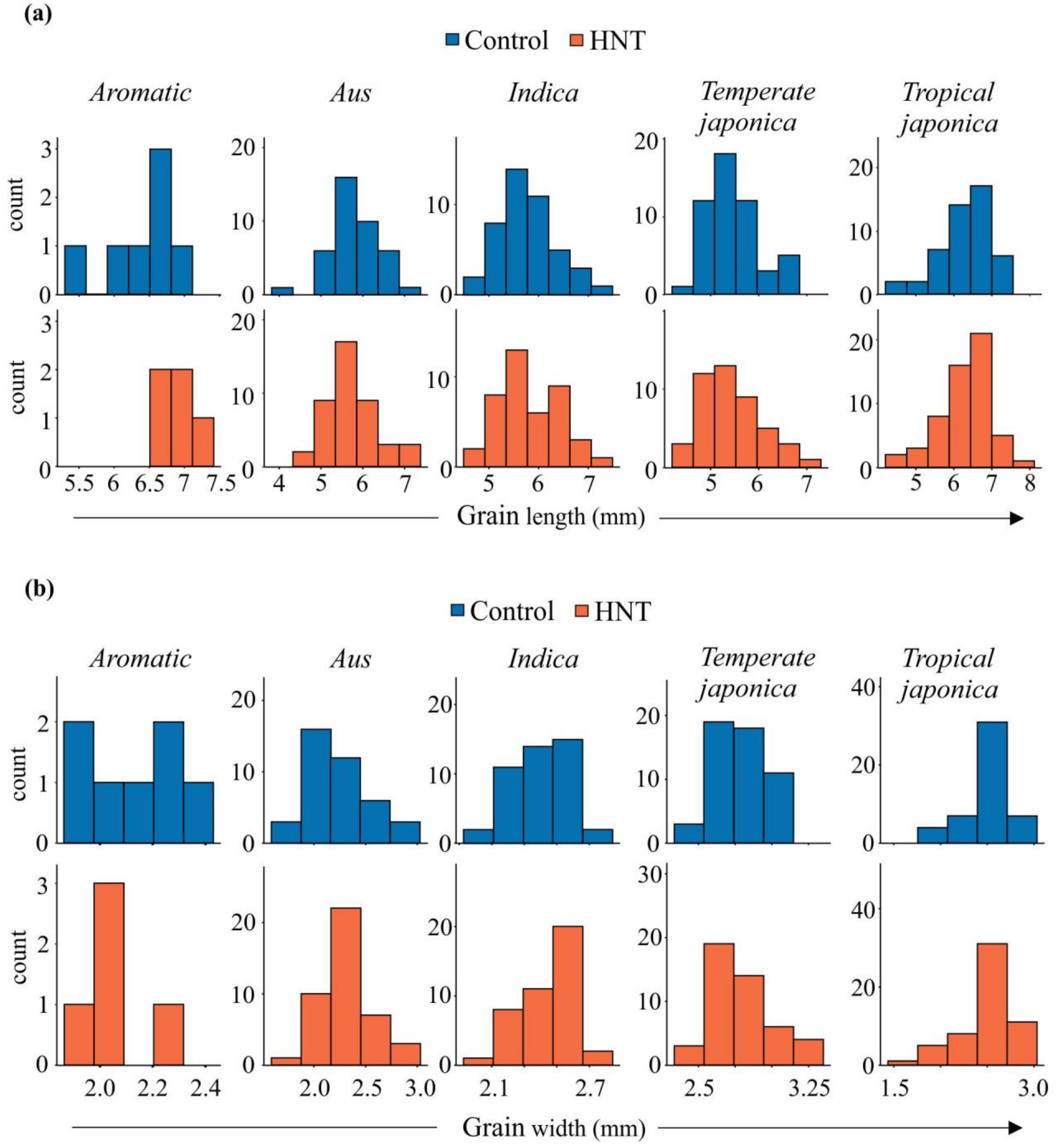

Figure S3: Sub-population level phenotypic distribution of mature grain length (a) and width (b) under control and high night temperature (HNT) stress.

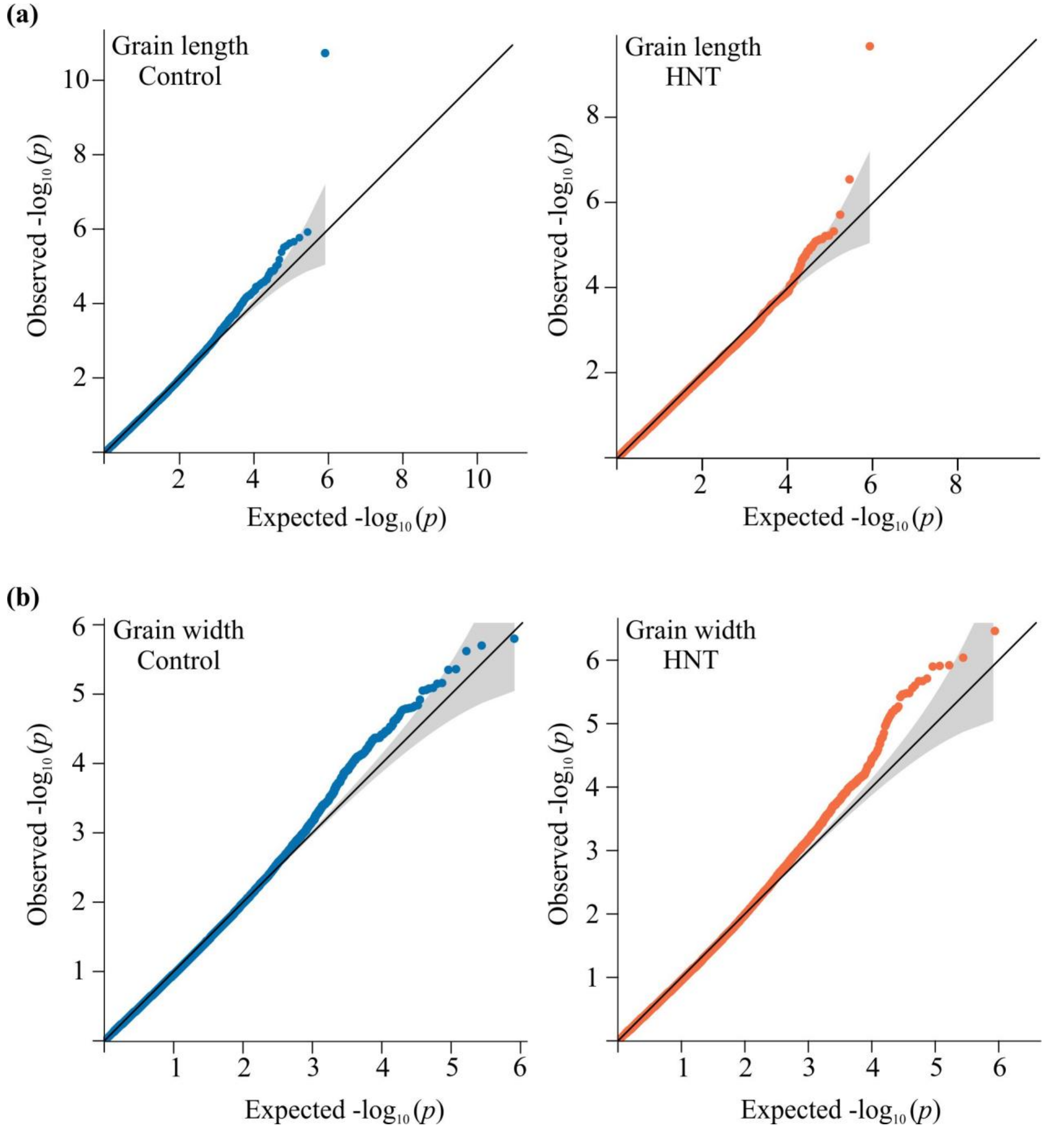

Figure S4. Q-Q plots of -log 10 (*p*) values obtained from the linear mixed model for mature grain length (a) and width (b) under control and high night temperature (HNT) stress.

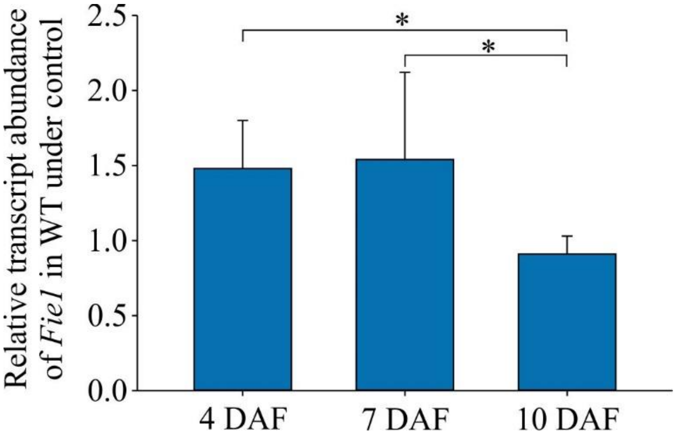

Figure S5. Relative transcript abundance of *Fie1* in wild type (WT) developing seeds (4, 7, and 10 days after fertilization; DAF) under control conditions. Values were normalized against 10 DAF. Error bars represent ± SD. For statistics, *t*-test was used, * $P < 0.05$.

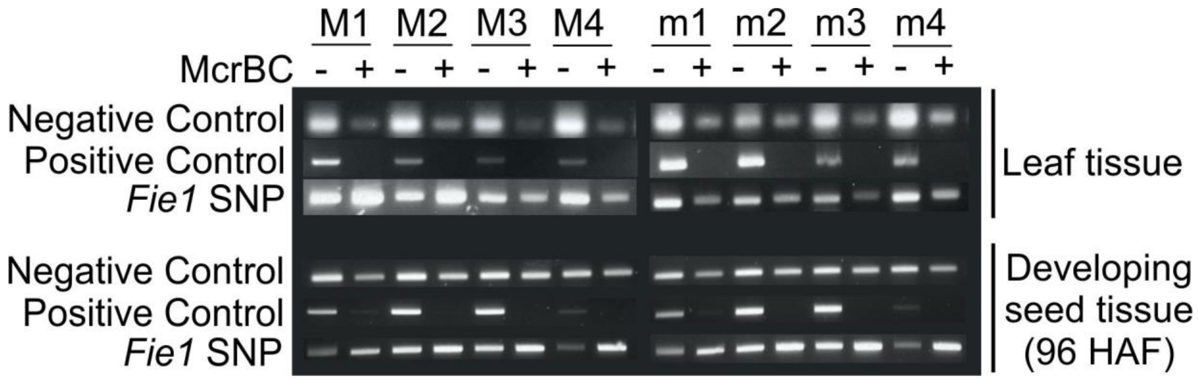

Figure S6. DNA methylation analysis. Primers used for McrBC assay encompassed the *Fie1* SNP in leaf and developing seed tissue (96 HAF). Negative and positive controls were used as previously discussed in Folsom *et al*., 2016. HAF: hours after fertilization, + represents McrBC digested DNA, – represents undigested DNA.

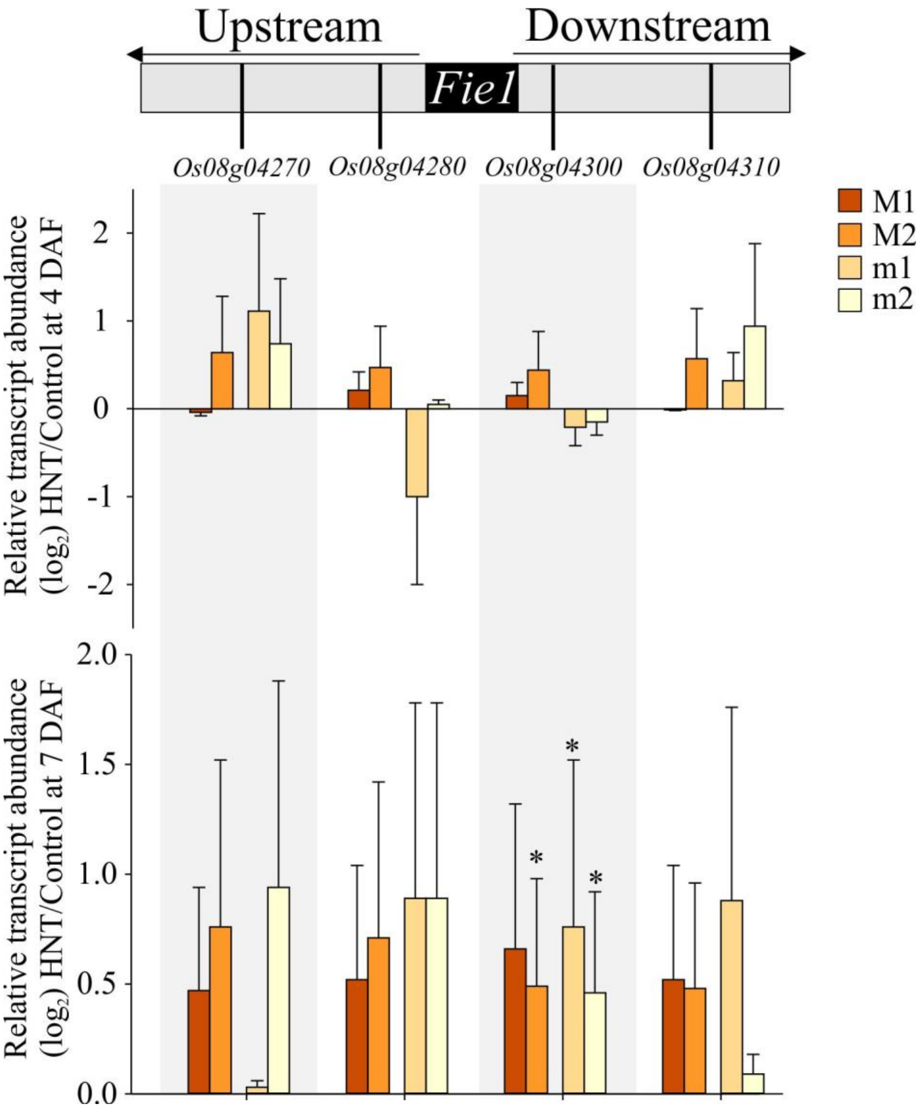

Figure S7. Relative transcript abundance of *Fie1* neighboring genes (two upstream and two downstream: upper panel) in developing seeds (4 and DAF; middle and lower panel) in two major (M1 and M2) and two minor (m1 and m2) allelic accessions under HNT. Values are normalized against control of the respective accession and developmental time-point. Values were normalized against 10 DAF. Error bars represent ± SD. For statistics, *t*-test was used, * $P < 0.05$.

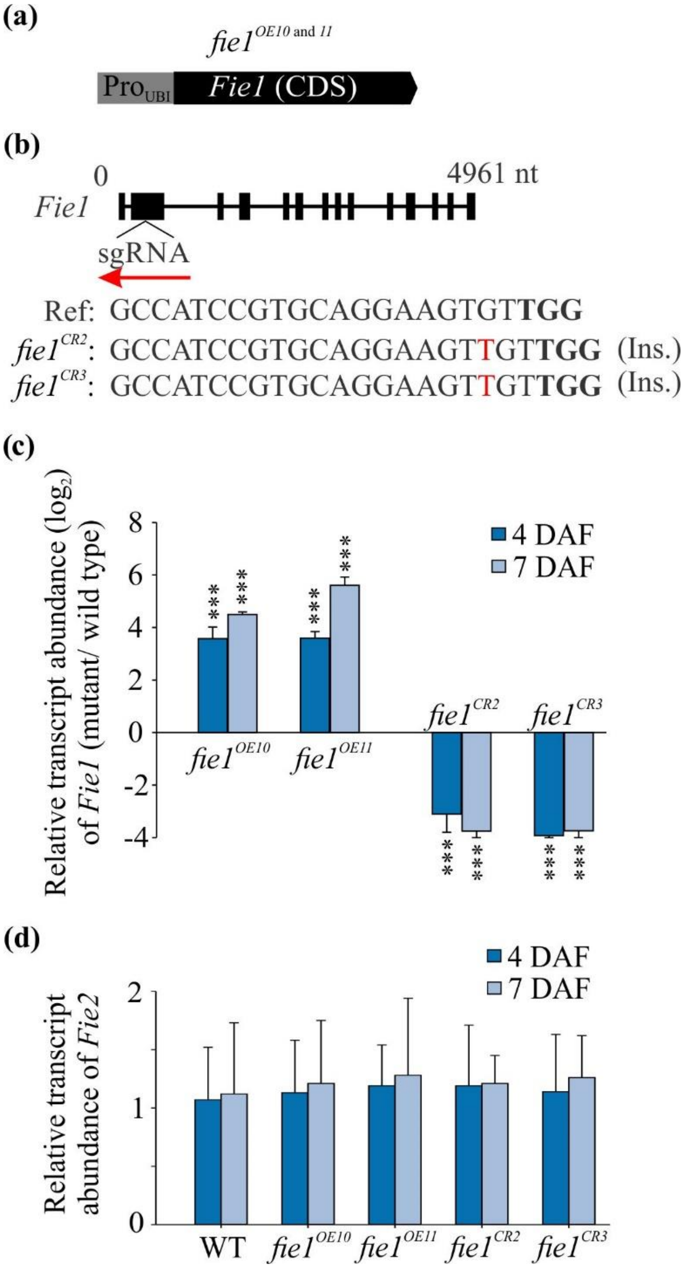

Figure S8. *Fie1* overexpression (a; *fie1*^{OE10} and *fie1*^{OE11}) and knockouts (b; *fie1*^{CR2} and *fie1*^{CR3}). Gene structure of *Fie1* depicting position, orientation (with red arrow), and sequences of sgRNAs designed to develop CRISPR-Cas9 mediated knockout mutants. Base pair change in homozygous mutants is represented by red letters, protospacer adjacent motif (PAM) region is shown in bold, and type of mutation is depicted in parentheses (Ref.: reference, Ins.: insertion). (c) Relative transcript abundance (log_2) of *Fie1* in mutants at 4 and 7 days after fertilization (DAF). Values were normalized against wild type for the respective developmental time-point. (d) Relative transcript abundance of *Fie2* in *Fie1* mutants at 4 and 7 DAF. Values are normalized against wild type for the respective developmental time-point. Error bars represent ± SD. For statistics in (c) and (d), *t*-test was used, *** indicates P <0.001. Error bars in (c) and (d) represent standard deviation.

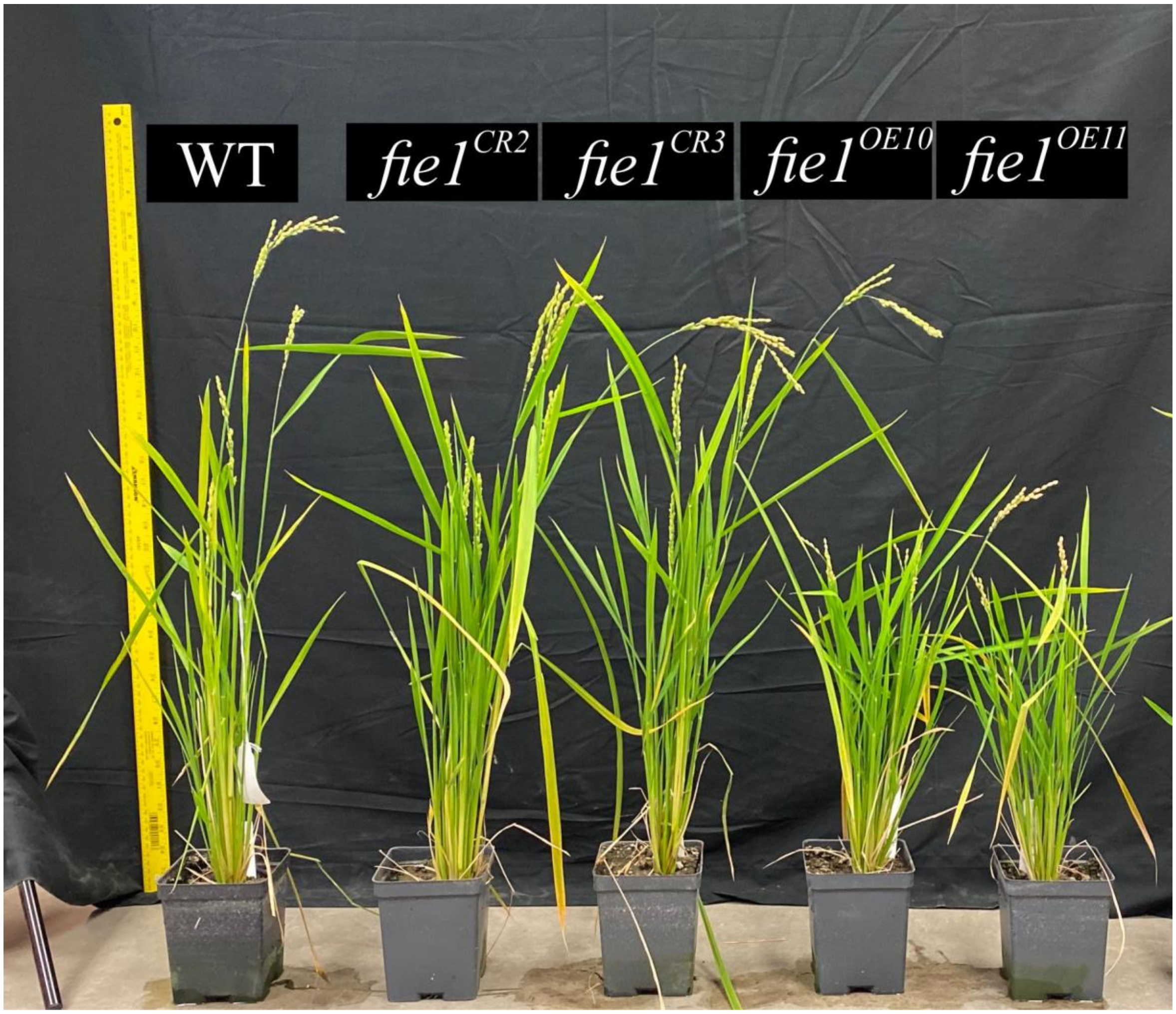

Figure S9. Representative images of wild type (WT), knockout ($fie1^{CR2}$ and $fie1^{CR3}$), and overexpression ($fie1^{OE10}$ and $fie1^{OE11}$) mutants at day 65.

(a)

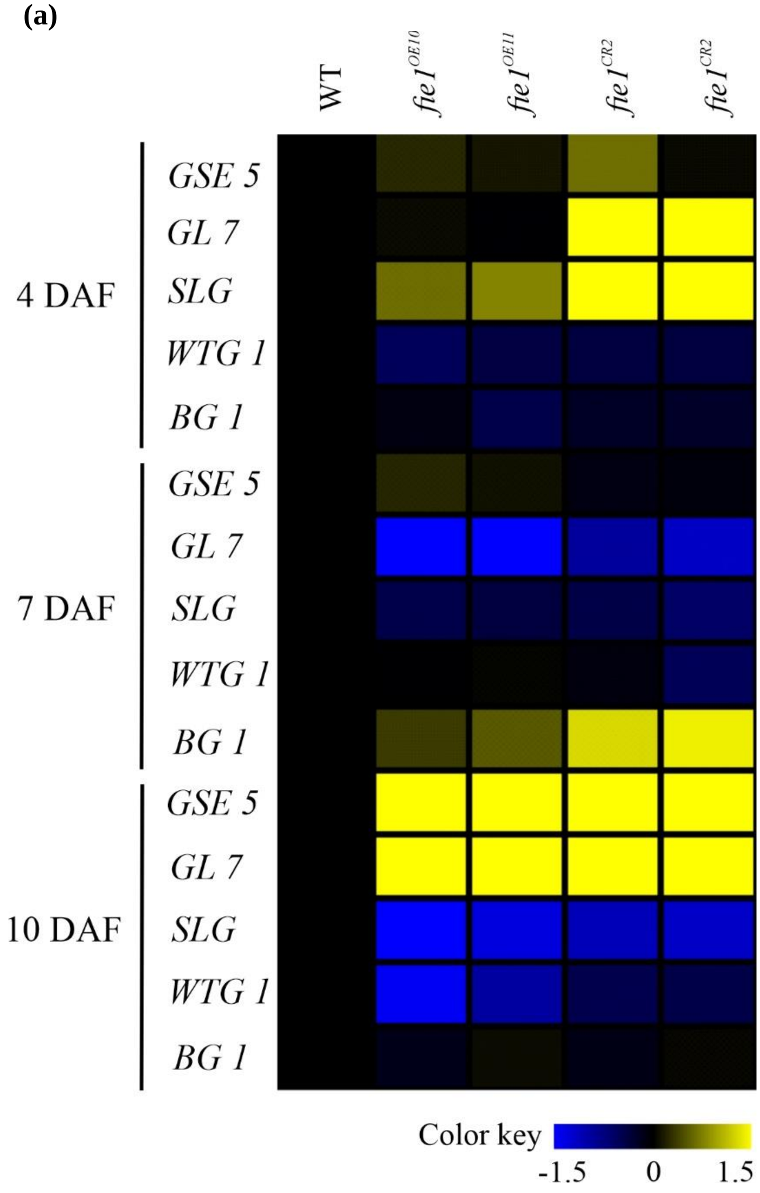
WT
fie1^OE10
fie1^OE11
fie1^CR2
fie1^CR2
4 DAF
7 DAF
10 DAF
GSE 5
GL 7
SLG
WTG 1
BG 1
Color key
-1.5
0
1.5

(b)

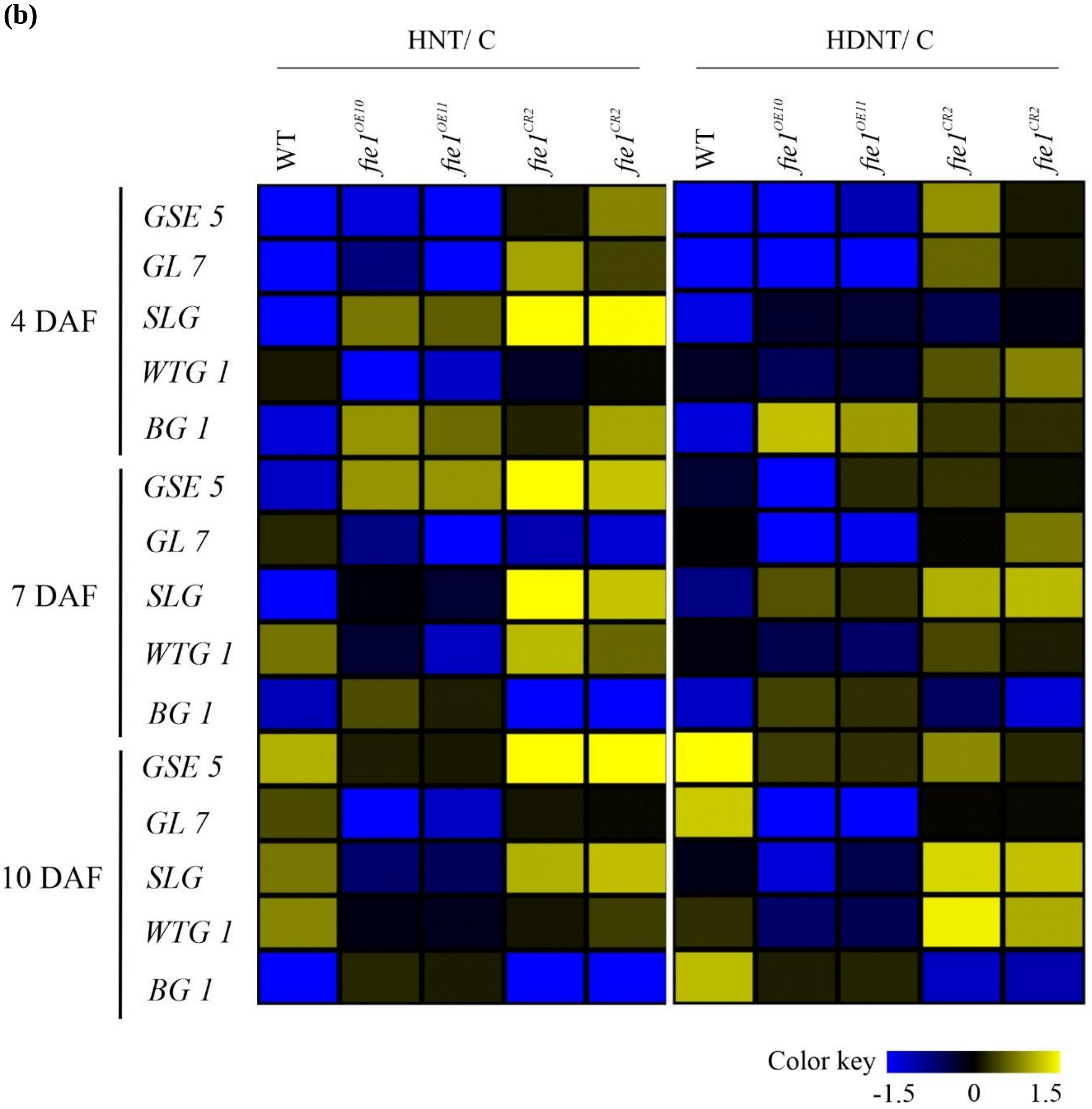

Figure S10. Gene expression analysis of grain size related genes. a) Heat map represents RT-qPCR analysis for selected set of grain size related genes under control conditions in the mutants (overexpression: *Fie1*^{OE10} and *Fie1*^{OE11} and knockouts: *fie1*^{CR2} and *fie1*^{CR3}) at 4, 7, and 10 days after fertilization (DAF). Values represent relative transcript abundance (log_2) normalized against WT for the respective developmental time-point. (b) Heat map represents RT-qPCR results for selected set of grain size related genes under HNT (left panel) and HDNT (right panel) in WT and mutants at 4, 7, and 10 DAF. Values represent relative transcript abundance (log_2) normalized against control for the respective plant line and developmental time-point.

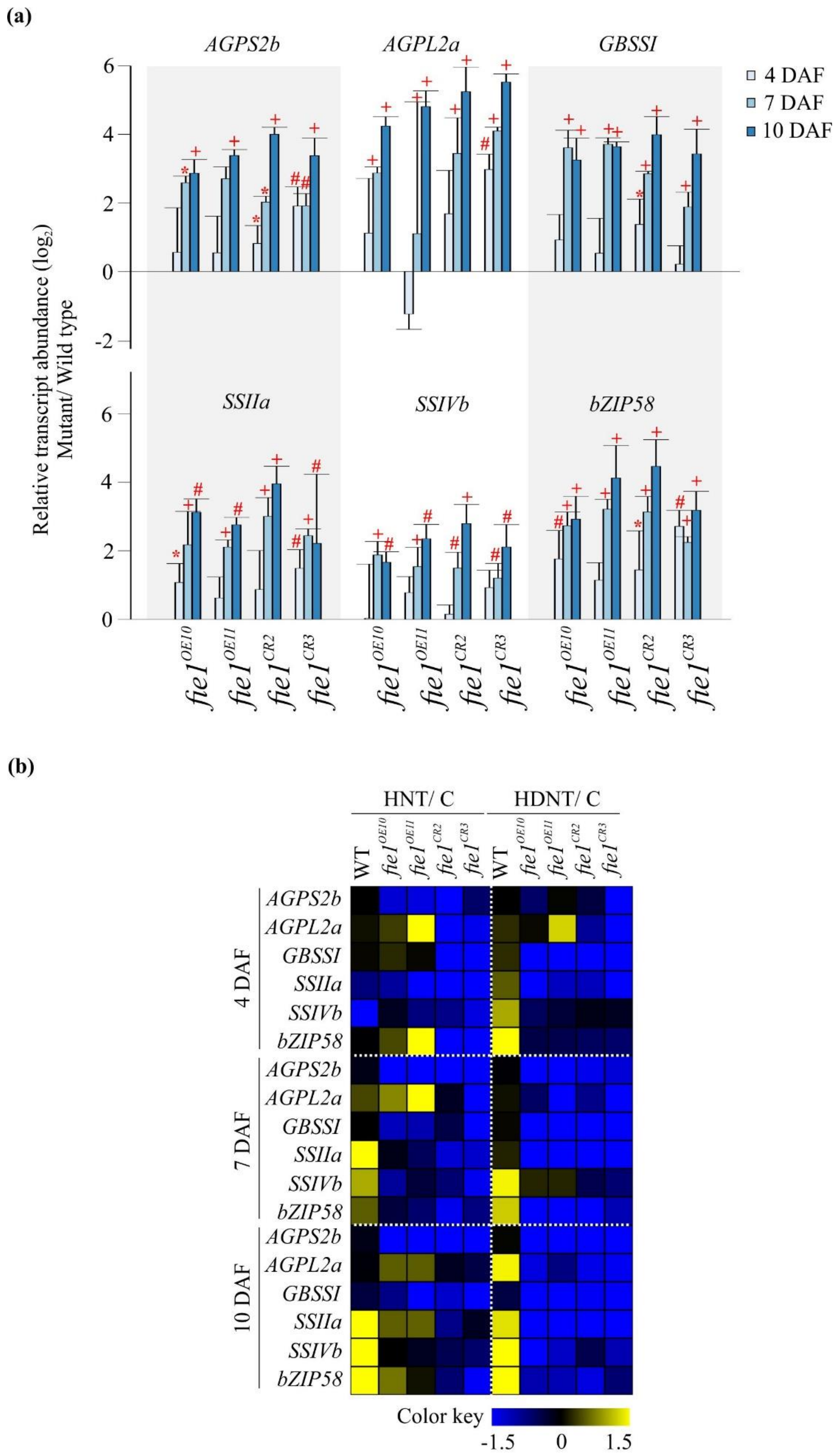

(a)
AGPS2b
AGPL2a
GBSSI
SSIIa
SSIVb
bZIP58
4 DAF
7 DAF
10 DAF
Relative transcript abundance (log2) Mutant/ Wild type
fie1^OE10
fie1^OE11
fie1^CR2
fie1^CR3
(b)
HNT/ C
HDNT/ C
WT
4 DAF
7 DAF
10 DAF
Color key
-1.5
0
1.5

Figure S11. (a) RT-qPCR analysis for selected set of starch biosynthesis genes under control conditions in the mutants (overexpression: $Fie1^{OE10}$ and $Fie1^{OE11}$ and knockouts: $fie1^{CR2}$ and $fie1^{CR3}$) at 4, 7, and 10 days after fertilization (DAF). Values represent relative transcript abundance (log_2) normalized against WT for the respective developmental time-point. Error bars represents ± SD For statistics, *t*-test was used. + indicates $P < 0.001$, 0.01, and 0.05 signified by +, #, and * respectively. (b) Heat map represents RT-qPCR results for selected set of starch biosynthesis genes under HNT and HDNT in WT and mutants at 4, 7, and 10 days after fertilization (DAF). Values represent relative transcript abundance (log_2) normalized against control for the respective plant line and developmental time-point.

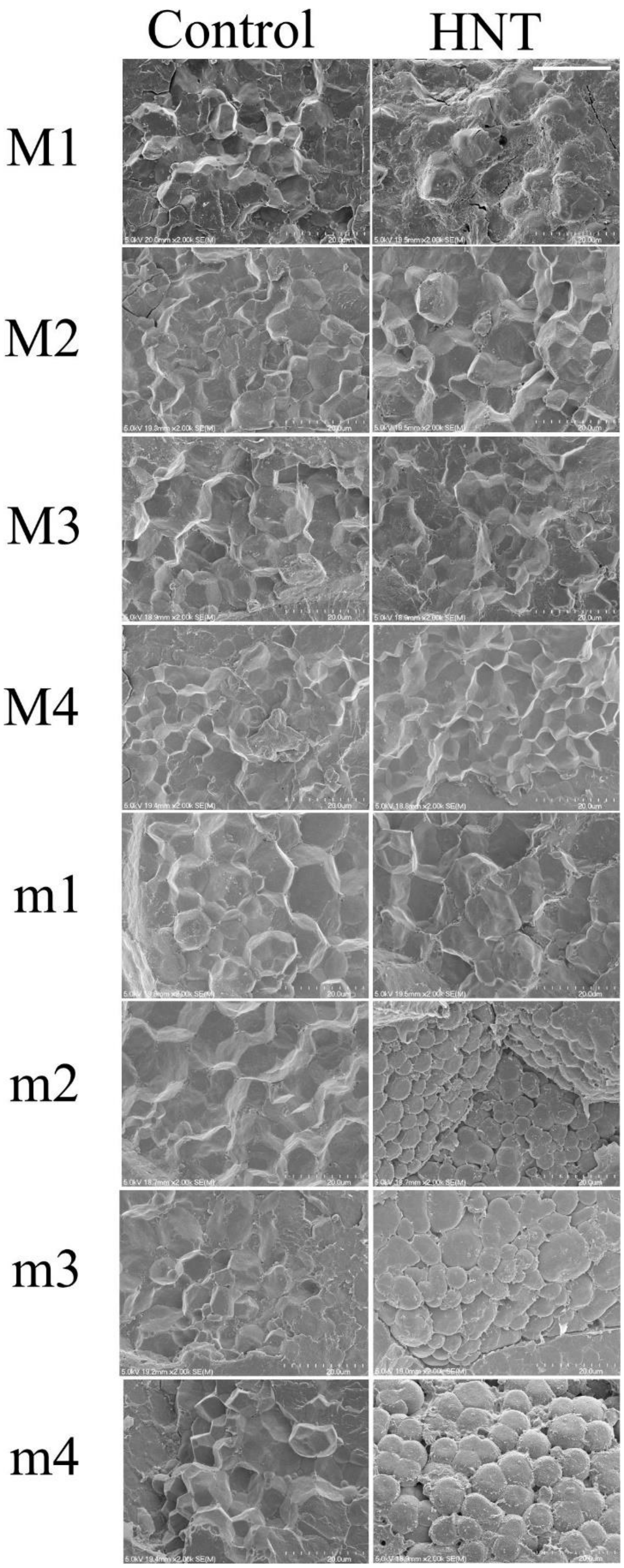

Figure S12. Cross-sections of mature grain from four major (M1 to M4) and four minor (m1 to m4) allelic accessions under HNT observed via scanning electron microscopy (scale: 20 μm).

References:

Folsom JJ, Begcy K, Hao X, Wang D, Walia H. **2014**. Rice fertilization-Independent Endosperm1 regulates seed size under heat stress by controlling early endosperm development. *Plant physiology* **165**: 238–48.
